# A trait dataset for Taiwan's breeding birds

**DOI:** 10.3897/BDJ.8.e49735

**Published:** 2020-05-19

**Authors:** Pei-Yu Tsai, Chie-Jen Ko, Chia Hsieh, Yi-Ting Su, Ya-Jung Lu, Ruey-Shing Lin, Mao-Ning Tuanmu

**Affiliations:** 1 Biodiversity Research Center, Academia Sinica, Taipei, Taiwan Biodiversity Research Center, Academia Sinica Taipei Taiwan; 2 Endemic Species Research Institute, Jiji, Nantou, Taiwan Endemic Species Research Institute Jiji, Nantou Taiwan; 3 Institute of Ecology and Evolutionary Biology, National Taiwan University, Taipei, Taiwan Institute of Ecology and Evolutionary Biology, National Taiwan University Taipei Taiwan; 4 BioSciences Department, Rice University, Houston, United States of America BioSciences Department, Rice University Houston United States of America; 5 Department of Life Sciences, National Cheng Kung University, Tainan, Taiwan Department of Life Sciences, National Cheng Kung University Tainan Taiwan; 6 Thematic Center for Systematics and Biodiversity Informatics, Biodiversity Research Center, Academia Sinica, Taipei, Taiwan Thematic Center for Systematics and Biodiversity Informatics, Biodiversity Research Center, Academia Sinica Taipei Taiwan

**Keywords:** breeding strategy, distribution, feeding behaviour, habitat use, life-history trait, morphology, movement, nest character

## Abstract

**Background:**

Species traits affect how a species interacts with the environment and other species and thus determine the role of the species in an ecosystem. They affect not only population dynamics of a species across space and over time, but also community structure and function through their key role in the community assembly processes. Information on species traits is also highly relevant for conservation planning as they determine the adaptive ability of a species in the face of environmental changes. However, information on species traits is usually scarce and sparsely distributed amongst diverse types of literature and sources. Difficulty in accessing comprehensive information on species traits has formed an essential knowledge gap, limiting our understanding of biodiversity patterns and ecosystem functioning and preventing effective conservation. Even for birds, a well-studied taxon, comprehensive trait information is still unavailable or distributed across different sources for many species.

**New information:**

In this study, we compiled information from a variety of sources on 23 traits for all breeding birds, including 157 resident and 14 summer visiting species, in Taiwan and surrounding islands. The 23 traits include those related to the movement patterns, morphology, geographic distributions, activity patterns, feeding behaviour, habitat use, and breeding behaviour and strategies of the species. The trait information was obtained, not only from published literature and datasets, but also from unpublished banding records and specimen measurements. The dataset also contains derived traits, including the elevation and temperature boundaries of species distribution ranges in Taiwan. In addition, structured information on nest characters, which is seldom compiled in trait datasets, has been made available, for the first time, for the breeding birds in Taiwan. Therefore, the most comprehensive trait dataset to date on breeding birds in Taiwan will allow trait-based research and applications in diverse topics and thus enhance our understanding of the patterns and dynamics of breeding bird diversity and its functions in Taiwan.

## Introduction

Traits are morphological, physiological, demographic and behaviour characteristics of organisms (McGill et al. 2006, Violle et al. 2007). They are related to organisms’ fitness, interactions with the environment and other organisms and ecological roles in an ecosystem (Reed et al. 2013, Kraft et al. 2015, Santini et al. 2016). They may also affect the community assembly process (Laureto et al. 2015) and thus determine the property of a community or even an ecosystem (McGill et al. 2006, Gross et al. 2017, Pigot et al. 2016). Moreover, traits are important in biodiversity conservation because they, with their movement, distributions, habitat use and breeding strategies, can be good indicators of species' adaptive ability and vulnerability to environmental changes (Pöyry et al. 2009, Pacifici et al. 2017) and species’ threatened status and extinction risk (Lee and Jetz 2010, Cooke et al. 2019).

Unfortunately, despite the importance of traits, comprehensive information on species traits is usually unavailable or is distributed across different sources, even for well-studied taxa, such as birds. EltonTraits (Wilman et al. 2014) is currently one of the most comprehensive (in terms of taxonomic coverage) trait datasets for birds. However, it lacks information on many important traits. For example, body mass is the only morphological trait in the EltonTraits dataset, although some other characteristics of morphology, such as tail length and wing shape, are related to the ecological role of a bird (Phillips et al. 2018, Hsu et al. 2013). In addition, the dataset also lacks information on the traits related to the breeding strategy, such as clutch size and nest structure, which critically determines the performance and fitness of birds (Griesser et al. 2007, DuRant et al. 2013, Altamirano et al. 2019), their adaptation capacity to changing environments (Deeming and Reynolds 2015, Pacifici et al. 2017) and their extinction risk (Cooke et al. 2019). Some other datasets cover more traits, but they are usually limited in terms of geographic and thus taxonomic coverage (eg. Garnett et al. 2015, Myhrvold et al. 2015), leaving many trait data gaps.

Taiwan has abundant biodiversity, including more than 600 bird species, thanks to the various climates and vegetation types along its almost 4000 m elevation gradient. Although bird traits have been found to be an important determinant of the diversity pattern and species composition of both native (Ding et al. 2005) and invasive birds in Taiwan (Su et al. 2016), a comprehensive dataset on bird traits is still lacking. Therefore, in this study, we compiled the information on 23 traits, including morphological, behaviour, distributional and life-history traits, for all breeding birds in Taiwan. This structured and relatively comprehensive trait dataset can support trait-based research and diverse applications on Taiwan's breeding birds. For example, several traits, related to thermal tolerance, diets and habitat use, have been associated with birds altitudinal migration tendencies to explore potential drivers of this behaviour (Tsai et al., in review). This dataset may also improve our ecological and evolutionary understanding of the community assembly in an island-like system (Mittelbach and Schemske 2015, Weber et al. 2017).

## General description

### Purpose

The aim of this study was to compile trait information from diverse sources and to provide a dataset containing structured and the most comprehensive trait information to date for all breeding birds in Taiwan.

### Additional information

We obtained information on 23 traits for Taiwan's 171 breeding bird species from seven sources (Table 1):

1. The Checklist of Birds of Taiwan issued by Chinese Wild Bird Federation (CWBF; CWBF 2017);
2. The Avifauna of Taiwan (Severinghaus et al. 2010);
3. The Handbook of the Birds of the World Alive (HBW Alive; Del Hoyo et al. 2018);
4. Banding data collected from 2008 to 2015 by the Monitoring Avian Productivity and Survivorship Program in Taiwan (MAPS, https://sites.google.com/birds-tesri.twbbs.org/maps-taiwan/);
5. Morphological measurements of the specimens from museums, including the Biodiversity Research Museum of Academia Sinica, National Taiwan Museum and Yamashina Institute for Ornithology;
6. The EltonTraits dataset (ver. 1.0; Wilman et al. 2014);
7. Our own calculations by overlapping bird occurrence records from eBird (Sullivan et al. 2009) and the environmental data layers of elevation (Global Digital Elevation Models GDEM; https://gisgeography.com/free-global-dem-data-sources) and temperatures (Climatologies at High Resolution for the Earth’s Land Surface Areas CHELSA; Karger et al. 2017).

Below are the detailed approaches for obtaining and compiling the information on individual traits:

**Movement**

One trait, residency, belongs to the category of movement. According to the Checklist of Birds of Taiwan (CWBF 2017), the residency of birds can be classified into six types: residents, summer visitors, winter visitors, passage migrants, straggers and introduced species. Although our dataset focuses on the trait information for the resident and summer visitor species in Taiwan, some of those species also contain winter visiting or passage migratory populations. Therefore, we used four binary variables to indicate whether a species contains a population of each of the four residency types (Table 2).

**Morphology**

Our dataset contains six morphological traits, including body mass, body length, head length, natural wing length, tail length and tarsus length (Table 1). We used three methods to calculate or obtain the mean and/or standard deviation of each of the traits: (1) calculating the two statistics from measurements on individual birds, (2) calculating the mean as the average of the maximum and minimum values reported in the data sources and (3) obtaining the two statistics directly from the data sources. The first method was to calculate the values from the measurements on living individuals or specimens, which were obtained from the Avifauna of Taiwan, MAPS banding datasets and the museum specimens. The measurements of juveniles and subadults were excluded from the calculation if the information was available. Some clear outliers (e.g. having a different order of magnitude) were also excluded. When measurements on individuals were not available from the above sources, we calculated the average of the maximum and minimum trait values reported in the Avifauna of Taiwan or HBW Alive. When only the mean and/or standard deviation were available in data sources, including the Avifauna of Taiwan, HBW Alive and the EltonTraits dataset, we included the statistics directly. The information on the data source, sample size and calculation method for each morphological trait of each species is provided in Table 3. For the measurements on individual birds, the information on the individual identifiers (identifiers of museum specimens or series number of banding records) is provided in Suppl. material 1. For the measurements obtained from the Avifauna of Taiwan, the volume and the page where the measurements were extracted are also included in the file.

**Distribution**

Four traits, lower and upper elevation boundaries and lower and upper temperature boundaries, are under the category of distribution (Table 1). To estimate the boundaries, we obtained the occurrence records of all the 171 species from the eBird database (downloaded on 16 March 2018; Sullivan et al. 2009) and used only the records collected after the year of 2000 within the study area. We obtained the elevation for each of the occurrence locations, based on the data layer from the ASTER Global Digital Elevation Models (v2; https://search.earthdata.nasa.gov/search/). To account for the uncertainty in GPS locating, we calculated the mean elevation within 100 m of each location. We then calculated the 2.5th and the 97.5th percentiles of the mean elevation values for each species as the lower and upper elevation boundaries, respectively, of the distribution range of the species in Taiwan. For the temperature boundaries of species distribution ranges, we obtained the temperature values for the same occurrence dataset, based on the time series data layers of the Climatologies at High Resolution for the Earth’s Land Surface Areas (http://chelsa-climate.org/; Karger et al. 2017). For each occurrence record, we obtained the mean temperature of the month when the record was collected in the field. We then calculated the 2.5th and the 97.5th percentiles of the mean values for each species as the lower and upper temperature boundaries, respectively. Due to the limited availability of the time series temperature data, we only used the occurrence records collected between 2000 and 2013 for this calculation.

**Activity and Feeding**

We obtained the information on the activity time, diet and foraging stratum of the bird species from the EltonTraits dataset (Wilman et al. 2014) by subsetting the dataset for the 171 breeding bird species in Taiwan. Therefore, the information on the three traits in our dataset is the same as that in the EltonTraits dataset. The reason for including the information was for completeness. The original information sources and the approaches for compiling the information can be found in Wilman et al. (2014).

**Habitat**

We obtained information on habitat use of the bird species from the Avifauna of Taiwan (Severinghaus et al. 2010) and defined the habitat types following the habitats classification scheme used by the Red List of Threatened Species of the International Union for Conservation of Nature (IUCN; https://www.iucnredlist.org/resources/habitat-classification-scheme). Thirteen of the 18 IUCN first-level habitat types were used by the 171 breeding bird species in Taiwan and, thus, 13 binary variables were included in our dataset to indicate which habitat types were used by each of the species (Table 2).

**Breeding**

Eight traits, including the clutch size, incubation period, egg length, egg width, brood parasitism, nest structure, nest site and nest attachment, are in the trait category of breeding (Table 1). The mean values of the clutch size and incubation period were calculated for each species from the measurements on individual broods reported in the Avifauna of Taiwan (Severinghaus et al. 2010), while the mean values of the egg length and egg width were from the measurements on individual eggs. When the measurements on individual broods or eggs were not available, we calculated the mean of the maximum and minimum values reported in the Avifauna of Taiwan (Severinghaus et al. 2010) or the HBW Alive (Del Hoyo et al. 2018). The information on the data source, sample size and calculation method for the four traits of each species is provided in Table 3.

We obtained the information on the nest structure, nest site and nest attachment approaches from the HBW Alive (Del Hoyo et al. 2018) and defined seven, seven and four types of the three traits (Table 1), respectively, following Fang et al. (2018). As a single species may build nests with more than one type of structure, site and/or attachment, we used seven, seven and four binary variables to indicate the types of the three traits, respectively, for each species (Table 2). Some bird species do not build their own nests, but lay their eggs in the nests of other species and rely on the hosts to raise their offspring. Therefore, we included another trait, brood parasitism, to indicate the brood parasite species, whose nest traits cannot be defined.

## Geographic coverage

### Description

**Figure 1.**
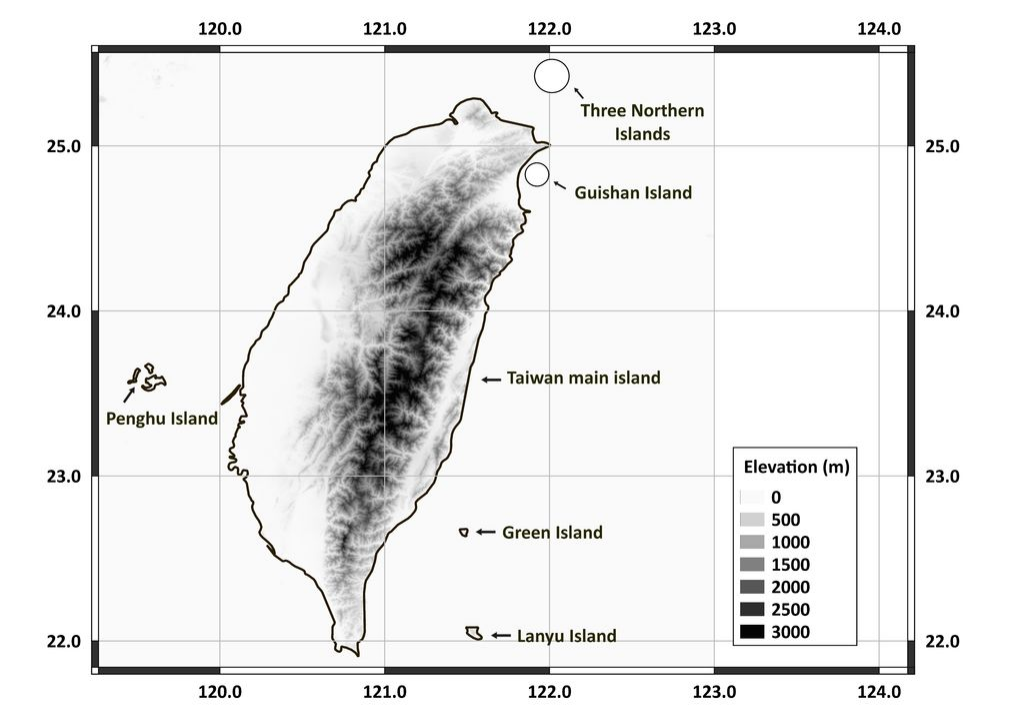
The location and elevation of Taiwan and surrounding islands.

Taiwan, including Taiwan's main island, Penghu Islands, Green Island, Lanyu Island, Guishan Island and the Three Northern Islands (Fig. 1).

### Coordinates

21°01'03 and 25°37'46" Latitude; 118°60'03 and 122°04'17" Longitude.

## Taxonomic coverage

### Description

This dataset contains trait information for all breeding birds in Taiwan, including 157 resident and 14 summer visitor bird species (CWBF; version of the Year 2017). These species belong to 15 orders and 58 families. We followed the HBW and BirdLife Taxonomic Checklist (http://datazone.birdlife.org/species/taxonomy; HBW-BirdLife Version 4.0) for nomenclature.

### Taxa included

**Table:** 

Rank	Scientific Name	Common Name
species	*Aix galericulata*	Mandarin Duck
species	*Anas zonorhyncha*	Chinese Spot-billed Duck
species	*Synoicus chinensis*	Asian Blue Quail
species	*Arborophila crudigularis*	Taiwan Partridge
species	*Bambusicola sonorivox*	Taiwan Bamboo-Partridge
species	*Syrmaticus mikado*	Mikado Pheasant
species	*Phasianus colchicus*	Common Pheasant
species	*Lophura swinhoii*	Swinhoe's Pheasant
species	*Tachybaptus ruficollis*	Little Grebe
species	*Ixobrychus sinensis*	Yellow Bittern
species	*Ixobrychus cinnamomeus*	Cinnamon Bittern
species	*Ardea purpurea*	Purple Heron
species	*Ardea alba*	Great White Egret
species	*Egretta garzetta*	Little Egret
species	*Egretta sacra*	Pacific Reef-Heron
species	*Bubulcus ibis*	Cattle Egret
species	*Butorides striata*	Green-backed Heron
species	*Nycticorax nycticorax*	Black-crowned Night-heron
species	*Gorsachius melanolophus*	Malayan Night-Heron
species	*Elanus caeruleus*	Black-shouldered Kite
species	*Pernis ptilorhynchus*	Oriental Honey-buzzard
species	*Spilornis cheela*	Crested Serpent-eagle
species	*Nisaetus nipalensis*	Mountain Hawk-eagle
species	*Ictinaetus malaiensis*	Black Eagle
species	*Accipiter trivirgatus*	Crested Goshawk
species	*Accipiter virgatus*	Besra
species	*Milvus migrans*	Black Kite
species	*Rallina eurizonoides*	Slaty-legged Crake
species	*Lewinia striata*	Slaty-breasted Rail
species	*Amaurornis phoenicurus*	White-breasted Waterhen
species	*Zapornia fusca*	Ruddy-breasted Crake
species	*Gallicrex cinerea*	Watercock
species	*Gallinula chloropus*	Common Moorhen
species	*Himantopus himantopus*	Himantopus himantopus
species	*Charadrius alexandrinus*	Kentish Plover
species	*Charadrius dubius*	Little Ringed Plover
species	*Rostratula benghalensis*	Greater Painted-snipe
species	*Hydrophasianus chirurgus*	Pheasant-tailed Jacana
species	*Turnix sylvaticus*	Common Buttonquail
species	*Turnix suscitator*	Barred Buttonquail
species	*Glareola maldivarum*	Oriental Pratincole
species	*Anous stolidus*	Brown Noddy
species	*Onychoprion anaethetus*	Bridled Tern
species	*Sternula albifrons*	Little Tern
species	*Sterna dougallii*	Roseate Tern
species	*Onychoprion fuscatus*	Sooty Tern
species	*Sterna sumatrana*	Black-naped Tern
species	*Thalasseus bergii*	Great Crested Tern
species	*Thalasseus bernsteini*	Chinese Crested Tern
species	*Columba pulchricollis*	Ashy WoodPigeon
species	*Streptopelia orientalis*	Oriental Turtle-Dove
species	*Streptopelia tranquebarica*	Red Turtle-dove
species	*Spilopelia chinensis*	Eastern Spotted Dove
species	*Macropygia tenuirostris*	Philippine Cuckoo-Dove
species	*Chalcophaps indica*	Grey-capped Emerald Dove
species	*Treron sieboldii*	White-bellied Pigeon
species	*Treron formosae*	Taiwan Green-pigeon
species	*Ramphiculus leclancheri*	Black-chinned Fruit-Dove
species	*Centropus bengalensis*	Lesser Coucal
species	*Hierococcyx sparverioides*	Large Hawk-cuckoo
species	*Cuculus saturatus*	Oriental Cuckoo
species	*Tyto longimembris*	Eastern Grass-owl
species	*Otus spilocephalus*	Mountain Scops-Owl
species	*Otus lettia*	Collared Scops-Owl
species	*Otus elegans*	Ryukyu Scops-Owl
species	*Ketupa flavipes*	Tawny Fish-Owl
species	*Glaucidium brodiei*	Collared Owlet
species	*Strix leptogrammica*	Brown Wood-Owl
species	*Strix nivicolum*	Himalayan Owl
species	*Ninox japonica*	Northern Boobook
species	*Caprimulgus affinis*	Savanna Nightjar
species	*Hirundapus cochinchinensis*	Silver-backed Needletail
species	*Apus nipalensis*	House Swift
species	*Alcedo atthis*	Common Kingfisher
species	*Psilopogon nuchalis*	Taiwan Barbet
species	*Picoides canicapillus*	Gray-capped Woodpecker
species	*Dendrocopos leucotos*	White-backed Woodpecker
species	*Picus canus*	Gray-faced Woodpecker
species	*Falco peregrinus*	Peregrine Falcon
species	*Pitta nympha*	Fairy Pitta
species	*Pericrocotus solaris*	Gray-chinned Minivet
species	*Coracina macei*	Indian Cuckooshrike
species	*Lanius schach*	Long-tailed Shrike
species	*Erpornis zantholeuca*	White-bellied Erpornis
species	*Oriolus chinensis*	Black-naped Oriole
species	*Oriolus traillii*	Maroon Oriole
species	*Dicrurus macrocercus*	Black Drongo
species	*Dicrurus aeneus*	Bronzed Drongo
species	*Hypothymis azurea*	Black-naped Monarch
species	*Terpsiphone atrocaudata*	Japanese Paradise-Flycatcher
species	*Garrulus glandarius*	Eurasian Jay
species	*Urocissa caerulea*	Taiwan Blue Magpie
species	*Dendrocitta formosae*	Gray Treepie
species	*Nucifraga caryocatactes*	Eurasian Nutcracker
species	*Corvus macrorhynchos*	Large-billed Crow
species	*Alauda gulgula*	Oriental Skylark
species	*Riparia chinensis*	Asian Plain Martin
species	*Hirundo rustica*	Barn Swallow
species	*Hirundo tahitica*	Tahiti Swallow
species	*Cecropis daurica*	Striated Swallow
species	*Delichon dasypus*	Asian House-Martin
species	*Periparus ater*	Coal Tit
species	*Sittiparus castaneoventris*	Chestnut-bellied Tit
species	*Parus monticolus*	Green-backed Tit
species	*Machlolophus holsti*	Yellow Tit
species	*Aegithalos concinnus*	Black-throated Tit
species	*Sitta europaea*	Eurasian Nuthatch
species	*Troglodytes troglodytes*	Northern Wren
species	*Cinclus pallasii*	Brown Dipper
species	*Spizixos semitorques*	Collared Finchbill
species	*Pycnonotus taivanus*	Styan's Bulbul
species	*Pycnonotus sinensis*	Light-vented Bulbul
species	*Hypsipetes leucocephalus*	Black Bulbul
species	*Hypsipetes amaurotis*	Brown-eared Bulbul
species	*Regulus goodfellowi*	Flamecrest
species	*Pnoepyga formosana*	Taiwan Cupwing
species	*Abroscopus albogularis*	Rufous-faced Warbler
species	*Horornis fortipes*	Brownish-flanked Bush-warbler
species	*Horornis acanthizoides*	Yellowish-bellied Bush-warbler
species	*Locustella alishanensis*	Taiwan Grasshopper-warbler
species	*Cisticola juncidis*	Zitting Cisticola
species	*Cisticola exilis*	Golden-headed Cisticola
species	*Prinia crinigera*	Striated Prinia
species	*Prinia flaviventris*	Yellow-bellied Prinia
species	*Prinia inornata*	Plain Prinia
species	*Fulvetta formosana*	Taiwan Fulvetta
species	*Sinosuthora webbiana*	Vinous-throated Parrotbill
species	*Suthora verreauxi*	Golden Parrotbill
species	*Yuhina brunneiceps*	Taiwan Yuhina
species	*Zosterops japonicus*	Japanese White-eye
species	*Zosterops meyeni*	Lowland White-eye
species	*Cyanoderma ruficeps*	Rufous-capped Babbler
species	*Pomatorhinus musicus*	Taiwan Scimitar-Babbler
species	*Erythrogenys erythrocnemis*	Black-necklaced Scimitar-Babbler
species	*Schoeniparus brunneus*	Dusky Fulvetta
species	*Alcippe morrisonia*	Grey-cheeked Fulvetta
species	*Garrulax taewanus*	Taiwan Hwamei
species	*Garrulax ruficeps*	Rufous-crowned Laughingthrush
species	*Garrulax poecilorhynchus*	Rusty Laughingthrush
species	*Trochalopteron morrisonianum*	White-whiskered Laughingthrush
species	*Heterophasia auricularis*	White-eared Sibia
species	*Liocichla steerii*	Taiwan Liocichla
species	*Sibia morrisoniana*	Taiwan Barwing
species	*Muscicapa ferruginea*	Ferruginous Flycatcher
species	*Niltava vivida*	Small Vivid Niltava
species	*Brachypteryx montana*	Javan Shortwing
species	*Myophonus insularis*	Taiwan Whistling-Thrush
species	*Enicurus scouleri*	Little Forktail
species	*Myiomela leucura*	White-tailed Blue Robin
species	*Tarsiger indicus*	White-browed Bush-Robin
species	*Tarsiger johnstoniae*	Collared Bush-Robin
species	*Ficedula hyperythra*	Snowy-browed Flycatcher
species	*Phoenicurus fuliginosus*	Plumbeous Redstart
species	*Monticola solitarius*	Blue Rock-Thrush
species	*Zoothera dauma*	Scaly Thrush
species	*Turdus mandarinus*	Chinese Blackbird
species	*Turdus poliocephalus*	Island Thrush
species	*Spodiopsar cineraceus*	White-cheeked Starling
species	*Acridotheres cristatellus*	Crested Myna
species	*Dicaeum minullum*	Plain Flowerpecker
species	*Dicaeum ignipectus*	Fire-breasted Flowerpecker
species	*Prunella collaris*	Alpine Accentor
species	*Motacilla alba*	White Wagtail
species	*Pyrrhula nipalensis*	Brown Bullfinch
species	*Pyrrhula erythaca*	Gray-headed Bullfinch
species	*Carpodacus formosanus*	Taiwan Rosefinch
species	*Passer cinnamomeus*	Russet Sparrow
species	*Passer montanus*	Eurasian Tree Sparrow
species	*Lonchura striata*	White-rumped Munia
species	*Lonchura punctulata*	Scaly-breasted Munia
species	*Lonchura atricapilla*	Chestnut Munia

## Traits coverage

This dataset contains 23 traits, grouped into 7 catogories (Table 1).

## Usage rights

### Use license

Other

### IP rights notes

Creative Commons Attribution (CC-BY) 4.0 License

## Data resources

### Data package title

A trait dataset for Taiwan's breeding birds

### Resource link

http://doi.org/10.5281/zenodo.3749452

### Number of data sets

3

### Data set 1.

#### Data set name

Taiwan_Breeding_Bird_Trait_ver2.csv

#### Data format

csv file

#### Number of columns

80

#### Download URL

http://doi.org/10.5281/zenodo.3749452

#### Description

Trait data table

**Data set 1.** 

Column label	Column description
SISRecID	Taxonomic identifier used by BirdLife International
ScientificName	Scientific name
ScientificName_CWBF	Scientific name used in the Checklist of Birds of Taiwan
Order	Order name
Family	Family name
CommName	Common name
Resident	Whether containing resident populations in Taiwan (0: no; 1: yes)
WinMig	Whether containing winter visitor populations in Taiwan (0: no; 1: yes)
SumMig	Whether containing summer visitor populations in Taiwan (0: no; 1: yes)
PasMig	Whether containing passage migratory populations in Taiwan (0: no; 1: yes)
BodyMassMean	Mean body mass (mg)
BodyMassSD	Standard deviation of body mass (mg)
BodyLengthMean	Mean body length (mm)
BodyLengthSD	Standard deviation of body length (mm)
HeadLengthMean	Mean head length (mm)
HeadLengthSD	Standard deviation of head length (mm)
WingLengthMean	Mean natural wing length (mm)
WingLengthSD	Standard deviation of natural wing length (mm)
TailLengthMean	Mean tail length (mm)
TailLengthSD	Standard deviation of tail length (mm)
TarsusLengthMean	Mean tarsus length (mm)
TarsusLengthSD	Standard deviation of tarsus length (mm)
LowElevBound	Lower elevation boundary of the distributional range in Taiwan (m)
HighElevBound	Upper elevation boundary of the distributional range in Taiwan (m)
LowTempBound	Lower temperature boundary of the distributional range in Taiwan (℃)
HighTempBound	Upper temperature boundary of the distributional range in Taiwan (℃)
Nocturnality	Whether being nocturnal (0: no; 1: yes)
Diet_inv	Percentage of invertebrates in the diet (%)
Diet_vend	Percentage of mammals and birds in the diet (%)
Diet_vect	Percentage of reptiles and amphibians in the diet (%)
Diet_fish	Percentage of fish in the diet (%)
Diet_vunk	Percentage of unknown vertebrates in the diet (%)
Diet_scav	Percentage of dead organisms in diet (%)
Diet_fruit	Percentage of fruits in diet (%)
Diet_nect	Percentage of nectar and pollen in diet (%)
Diet_seed	Percentage of seeds in diet (%)
Diet_planto	Percentage of other plant materials in diet (%)
ForStrat_watbelowsurf	Prevalence of foraging below water surfaces (%)
ForStrat_wataroundsurf	Prevalence of foraging around water surfaces (%)
ForStrat_ground	Prevalence of foraging on the ground (%)
ForStrat_understory	Prevalence of foraging below 2 m in understorey (%)
ForStrat_midhigh	Prevalence of foraging in mid to high levels in trees or high bushes (%)
ForStrat_canopy	Prevalence of foraging in or just above tree canopy (%)
ForStrat_aerial	Prevalence of foraging above vegetation or any structures (%)
Habitat_forest	Whether using forest as habitat (0: no; 1: yes)
Habitat_savanna	Whether using savannah as habitat (0: no; 1: yes)
Habitat_shrubland	Whether using shrubland as habitat (0: no; 1: yes)
Habitat_grassland	Whether using grassland as habitat (0: no; 1: yes)
Habitat_wetland	Whether using wetland as habitat (0: no; 1: yes)
Habitat_rocky	Whether using rocky areas as habitat (0: no; 1: yes)
Habitat_neritic	Whether using nertic areas as habitat (0: no; 1: yes)
Habitat_intertidal	Whether using intertidal areas as habitat (0: no; 1: yes)
Habitat_coastal_supratidal	Whether using coastal or supratidal areas as habitat (0: no; 1: yes)
Habitat_artificial_terrestrial	Whether using terrestrial artificial areas as habitat (0: no; 1: yes)
Habitat_artificial_aquatic	Whether using aquatic artificial areas as habitat (0: no; 1: yes)
Habitat_other	Whether using other habitat types (0: no; 1: yes)
Habitat_unknown	Whether using unknown habitat types (0: no; 1: yes)
ClutchSize	Mean clutch size
IncuPeriod	Mean incubation period (day)
EggLength	Mean length of the long diameter of an egg (mm)
EggWidth	Mean length of the short diameter of an egg (mm)
BroodParasite	Whether being a brood parasite (0: no; 1: yes)
NestStr_scrape	Whether using a scrape nest (0: no; 1: yes)
NestStr_platform	Whether using a platform nest (0: no; 1: yes)
NestStr_cup	Whether using a cup nest (0: no; 1: yes)
NestStr_dome	Whether using a domed nest with no tunnel (0: no; 1: yes)
NestStr_dome_tunnel	Whether using a domed nest with a tunnel (0: no; 1: yes)
NestStr_primary_cavity	Whether using a primary cavity nest (0: no; 1: yes)
NestStr_second_cavity	Whether using a secondary cavity nest (0: no; 1: yes)
NestSite_ground	Whether using a nest built on the ground (0: no; 1: yes)
NestSite_tree	Whether using a nest built in/on trees (0: no; 1: yes)
NestSite_non_tree	Whether using a nest built on non-tree vegetation (0: no; 1: yes)
NestSite_cliff	Whether using a nest built on cliff or banks (0: no; 1: yes)
NestSite_underground	Whether using a nest built underground (0: no; 1: yes)
NestSite_waterbody	Whether using a nest built in water bodies (0: no; 1: yes)
NestSite_termite	Whether using a nest built in termite or ant nests (0: no; 1: yes)
NestAtt_basal	Whether using a nest with basal attachment (0: no; 1: yes)
NestAtt_forked	Whether using a nest with horizontally-forked attachment (0: no; 1: yes)
NestAtt_lateral	Whether using a nest with lateral attachment (0: no; 1: yes)
NestAtt_pensile	Whether using a nest with pensile attachment (0: no; 1: yes)

### Data set 2.

#### Data set name

Column_descriptions.csv

#### Data format

csv file

#### Number of columns

5

#### Download URL

http://doi.org/10.5281/zenodo.3749452

#### Description

Descriptions for the columns in the trait data table

**Data set 2.** 

Column label	Column description
Column name	Name of the column
Trait: State	Names of the trait and trait state (if available), separated by a colon
Description	Description of the value in the column
Data type	Type of the data value (numeric, binary or character)
Units	Unit of the data value (if available)

### Data set 3.

#### Data set name

Data_sources.csv

#### Data format

csv file

#### Number of columns

7

#### Download URL

http://doi.org/10.5281/zenodo.3749452

#### Description

Data sources of individual data values used for calculating the values of six morphological and four breeding traits included in the data table. The number of data values and the calculation method are also provided.

**Data set 3.** 

Column label	Column description
SISRecID	Taxonomic identifier used by BirdLife International
ScientificName	Scientific name following the HBW/BirdLife International version 4.0
ScientificName_CWBF	Scientific name used in the Checklist of Birds of Taiwan
TraitName	Name of traits
SampleSize	Number of data values used for calculating the mean and/or sd of trait values
Method	Method for obtaining the statistics of traits
DataSource	Source of individual data values

## Supplementary Material

E5273339-5AFA-5CF6-9734-EB618257793B 10.3897/BDJ.8.e49735.suppl1 Supplementary material 1 Source of individual data values for six morphological and four breeding traits Data type Data source information Brief description The data sources of individual data values for each of the traits and for each species are provided in the "DataSource" column. Sources for individual data values for the same trait of the same species are separated by semicolons. Source information contains the data source and an individual identifier, which are separated by a dash. For the values extracted from the Avifauna of Taiwan, the source information includes the volume and page where the values were extracted and are shown following the prefix "Vol" and "P", respectively. File: oo_408736.csv https://binary.pensoft.net/file/408736 Mao-Ning Tuanmu

## Figures and Tables

**Table 1.** Definitions and information sources of the 23 traits included in the dataset

**Category**	**Trait**	**Definition**	Information source*
Movement	Residency	Four states: Resident: birds stay in Taiwan year-round Winter visitor: migratory birds which arrive in Taiwan in autumn, spend the wintertime in Taiwan and leave for their breeding grounds outside Taiwan in spring Summer visitor: migratory birds which arrive in Taiwan in spring, breed in Taiwan and leave for their wintering areas outside Taiwan at the end of summer or early autumn Passage migrants: migratory birds which pass through Taiwan during their migration, possibly stopping in Taiwan for a few days, but not staying for extended periods of time	(1)
Morphology	Body mass	Weight of a bird	(2), (3), (4), (5), (6)
	Body length	Distance from the tip of the bill to the end of the tail	
	Head length	Distance from the tip of the bill to the back of the skull	
	Natural wing length	Distance from the carpal joint to the tip of the longest primary feather	
	Tail length	Distance from the root to the end of the longest tail feather	
	Tarsus length	Distance from the base of the tarsus to the tip of the middle toe	
Distribution	Lower elevation boundary	Lower elevation boundary of the distribution range in Taiwan	(7)
	Upper elevation boundary	Upper elevation boundary of the distribution range in Taiwan	
	Lower temperature boundary	Lower temperature boundary of the distribution range in Taiwan	
	Upper temperature boundary	Upper temperature boundary of the distribution range in Taiwan	
Activity	Nocturnality	Birds foraging mainly at night	(6)
Feeding	Diet	Ten food types in the diet (Wilman et al. 2014): Invertebrates: invertebrates-general, aquatic invertebrates, shrimp, krill, squid, crustacaeans, molluscs, cephalapod, polychaetes, gastropods, orthoptera, terrestrial invertebrates, ground insects, insect larvae, worms, orthopterans, flying insects Mammals and birds Reptiles and amphibians: reptiles, snakes, amphibians, salamanders Fish Unknown vertebrates: vertebrates-general or unknown Dead organisms: garbage, offal, carcasses, trawlers, carrion Fruits: fruits, drupes Nectar and pollen: nectar, pollen, plant exudates, gums Seeds: seeds, maize, nuts, spores, wheat, grains Other plant parts: grass, ground vegetation, seedlings, weeds, lichen, moss, small plants, reeds, cultivated crops, forbs, vegetables, fungi, roots, tubers, legumes, bulbs, leaves, above-ground vegetation, twigs, bark, shrubs, herbs, shoots, aquatic vegetation, aquatic plants	(6)
	Foraging stratum	Seven foraging strata (Wilman et al. 2014): Below the water surface: foraging below the water surface Around the water surface: foraging on or just (<12.5 cm) below the water surface On the ground: foraging on the ground In forest understorey: foraging below 2 m in the understorey in a forest, forest edges, bushes or shrubs In middle to high levels in trees: foraging in mid- to high-levels in trees or high bushes (2 m upwards), but below the canopy In or above tree canopy: foraging in or just above (from) tree canopy In aerial: foraging well above vegetation or any structures	
Habitat	Habitat types	Thirteen habitat types (IUCN Red List of Threatened Species; http://www.iucnredlist.org/): Forest: areas with continuous stands of trees, including both forested and wooded areas Savannah: areas with grass ground cover with an overstorey of widely-spaced trees and shrubs Shrubland: areas with scrub, bushland and thicket Grassland: areas with grasses and broadleaved herbaceous plants, without or with very sparsely-distributed trees Wetland: areas with marsh or water, where the water is either flowing or stagnant Rocky area: including inland cliffs, mountain peaks, talus and feldmark Neritic area: including submergence, nearshore, on or over the continental shelf or oceanic island shelf Intertidal area: areas of the shore between the extremes of high and low tides Coastal or supratidal area: coastal areas above the high tide mark Terrestrial artificial area: human-made terrestial habitats Aquatic artificial area: human-made wetland habitats Other habitats Unknown habitat	(2)
Breeding	Clutch size	Number of eggs laid by a female bird in a single brood	(2), (3)
	Incubation period	Length of the period from laying the last egg of the clutch until egg hatching	
	Egg length	Length of the long diameter of eggs	
	Egg width	Length of the short diameter of eggs	
	Brood parasitism	A type of social parasitism in which eggs are laid in the nests of other birds and the young are reared by the hosts	
	Nest structure	Structure of a nest; including seven types (Fang et al. 2018): Scrape: a nest without obvious construction or with only brief scratching or cleaning Platform: a nest where feathers, leaves, sticks or vines are stacked or loosely intertwined to form a platform Cup: a nest with an erected, surrounding rim made by interweaving nest materials or mud; varying in depth but parental birds could not entirely hide inside the nest Dome: a nest where a parental bird could sit inside without exposing the body Dome with a tunnel: a domed nest with a tunnel exit Primary cavity: a nest built inside a cave excavated directly by the birds using the cave Secondary cavity: a nest built inside a cave generated naturally or originally excavated by other animals	
	Nest site	Location where a nest was built, including seven types (Fang et al. 2018): Ground: on the ground Tree: on/in trees Non-tree vegetation: on bushes, bamboo or thick tangled herbaceous vegetation Cliff and bank: in cliffs, riverbanks or piles of soil or rocks Underground: in burrows underground Waterbody: on the surface of water or piled up from the bottom of lakes or ponds and thus surrounded by water Termite/ant nest: in termite or ant nests	
	Nest attachment	The way a nest is attached to a supporting object, including four types (Fang et al. 2018): Basal: a nest supported mainly from its bottom Horizontally forked: a nest attached to two or more horizontally forked tree or bush branches by the lateral parts of the nest Lateral: a nest attached to a supporting object solely by its lateral parts Pensile: a nest hanged down from a supporting object	

* Please refer to the General Description for the details of the information sources

**Table 2.** Information on the columns of the dataset table

**Column name**	**Trait: State**	**Description**	**Data type**	**Units**
SISRecID	-	Taxonomic identifier used by BirdLife International	character	
ScientificName	-	Scientific name	character	
ScientificName_CWBF	-	Scientific name used in the Checklist of Birds of Taiwan	character	
Order	-	Order name	character	
Family	-	Family name	character	
CommName	-	Common name	character	
Resident	Residency: Resident	Whether containing resident populations in Taiwan	binary (0: no; 1: yes)	
WinMig	Residency: Winter visitor	Whether containing winter visitor populations in Taiwan	binary (0: no; 1: yes)	
SumMig	Residency: Summer visitor	Whether containing summer visitor populations in Taiwan	binary (0: no; 1: yes)	
PasMig	Residency: Passage migrator	Whether containing passage migratory populations in Taiwan	binary (0: no; 1: yes)	
BodyMassMean	Body mass: Mean	Mean body mass	numeric	mg
BodyMassSD	Body mass: Standard deviation	Standard deviation of body mass	numeric	mg
BodyLengthMean	Body length: Mean	Mean body length	numeric	mm
BodyLengthSD	Body length: Standard deviation	Standard deviation of body length	numeric	mm
HeadLengthMean	Head length: Mean	Mean head length	numeric	mm
HeadLengthSD	Head length: Standard deviation	Standard deviation of head length	numeric	mm
WingLengthMean	Natural wing length: Mean	Mean natural wing length	numeric	mm
WingLengthSD	Natural wing length: Standard deviation	Standard deviation of wing length	numeric	mm
TailLengthMean	Tail length: Mean	Mean tail length	numeric	mm
TailLengthSD	Tail length: Standard deviation	Standard deviation of tail length	numeric	mm
TarsusLengthMean	Tarsus length: Mean	Mean tarsus length	numeric	mm
TarsusLengthSD	Tarsus length: Standard deviation	Standard deviation of tarsus length	numeric	mm
LowElevBound	Lower elevation boundary	Lower elevation boundary of the distributional range in Taiwan	numeric	m
HighElevBound	Upper elevation boundary	Upper elevation boundary of the distributional range in Taiwan	numeric	m
LowTempBound	Lower temperature boundary	Lower temperature boundary of the distributional range in Taiwan	numeric	℃
HighTempBound	Upper temperature boundary	Upper temperature boundary of the distributional range in Taiwan	numeric	℃
Nocturnality	Nocturnality	Whether being nocturnal	binary (0: no; 1: yes)	
Diet_inv	Diet: Invertebrates	Percentage of invertebrates in the diet	numeric	%
Diet_vend	Diet: Mammals and birds	Percentage of mammals and birds in the diet	numeric	%
Diet_vect	Diet: Reptiles and amphibians	Percentage of reptiles and amphibians in the diet	numeric	%
Diet_fish	Diet: Fish	Percentage of fish in the diet	numeric	%
Diet_vunk	Diet: Unknown vertebrates	Percentage of unknown vertebrates in the diet	numeric	%
Diet_scav	Diet: Dead organisms	Percentage of dead organisms in diet	numeric	%
Diet_fruit	Diet: Fruits	Percentage of fruits in diet	numeric	%
Diet_nect	Diet: Nectar and pollen	Percentage of nectar and pollen in diet	numeric	%
Diet_seed	Diet: Seeds	Percentage of seeds in diet	numeric	%
Diet_planto	Diet: Other plant parts	Percentage of other plant materials in diet	numeric	%
ForStrat_watbelowsurf	Foraging stratum: Below the water surface	Prevalence of foraging below water surfaces	numeric	%
ForStrat_wataroundsurf	Foraging stratum: Around the water surface	Prevalence of foraging around water surfaces	numeric	%
ForStrat_ground	Foraging stratum: On the ground	Prevalence of foraging on the ground	numeric	%
ForStrat_understory	Foraging stratum: In forest understorey	Prevalence of foraging below 2 m in understorey	numeric	%
ForStrat_midhigh	Foraging stratum: In middle to high levels in trees	Prevalence of foraging in mid to high levels in trees or high bushes	numeric	%
ForStrat_canopy	Foraging stratum: In or above tree canopy	Prevalence of foraging in or just above tree canopy	numeric	%
ForStrat_aerial	Foraging stratum: In aerial	Prevalence of foraging above vegetation or any structures	numeric	%
Habitat_forest	Habitat type: Forest	Whether using forest as habitat	binary (0: no; 1: yes)	
Habitat_savanna	Habitat type: Savannah	Whether using savannah as habitat	binary (0: no; 1: yes)	
Habitat_shrubland	Habitat type: Shrubland	Whether using shrubland as habitat	binary (0: no; 1: yes)	
Habitat_grassland	Habitat type: Grassland	Whether using grassland as habitat	binary (0: no; 1: yes)	
Habitat_wetland	Habitat type: Wetland	Whether using wetland as habitat	binary (0: no; 1: yes)	
Habitat_rocky	Habitat type: Rocky area	Whether using rocky areas as habitat	binary (0: no; 1: yes)	
Habitat_neritic	Habitat type: Neritic area	Whether using nertic areas as habitat	binary (0: no; 1: yes)	
Habitat_intertidal	Habitat type: Intertidal area	Whether using intertidal areas as habitat	binary (0: no; 1: yes)	
Habitat_coastal_supratidal	Habitat type: Coastal or supratidal area	Whether using coastal or supratidal areas as habitat	binary (0: no; 1: yes)	
Habitat_artificial_terrestrial	Habitat type: Terrestrial artificial area	Whether using terrestrial artificial areas as habitat	binary (0: no; 1: yes)	
Habitat_artificial_aquatic	Habitat type: Aquatic artificial area	Whether using aquatic artificial areas as habitat	binary (0: no; 1: yes)	
Habitat_other	Habitat type: Other habitats	Whether using other habitat types	binary (0: no; 1: yes)	
Habitat_unknown	Habitat type: Unknown habitat	Whether using unknown habitat types	binary (0: no; 1: yes)	
ClutchSize	Clutch size	Mean clutch size	numeric	
IncuPeriod	Incubation period	Mean incubation period	numeric	day
EggLength	Egg length	Mean length of the long diameter of an egg	numeric	mm
EggWidth	Egg width	Mean length of the short diameter of an egg	numeric	mm
BroodParasite	Brood parasitism	Whether being a brood parasite	binary (0: no; 1: yes)	
NestStr_scrape	Nest structure: Scrape	Whether using a scrape nest	binary (0: no; 1: yes)	
NestStr_platform	Nest structure: Platform	Whether using a platform nest	binary (0: no; 1: yes)	
NestStr_cup	Nest structure: Cup	Whether using a cup nest	binary (0: no; 1: yes)	
NestStr_dome	Nest structure: Dome	Whether using a domed nest with no tunnel	binary (0: no; 1: yes)	
NestStr_dome_tunnel	Nest structure: Dome with tunnel	Whether using a domed nest with a tunnel	binary (0: no; 1: yes)	
NestStr_primary_cavity	Nest structure: Primary cavity	Whether using a primary cavity nest	binary (0: no; 1: yes)	
NestStr_second_cavity	Nest structure: Secondary cavity	Whether using a secondary cavity nest	binary (0: no; 1: yes)	
NestSite_ground	Nest Site: Ground	Whether using a nest built on the ground	binary (0: no; 1: yes)	
NestSite_tree	Nest Site: Tree	Whether using a nest built in/on trees	binary (0: no; 1: yes)	
NestSite_non_tree	Nest Site: Non-tree vegetation	Whether using a nest built on non-tree vegetation	binary (0: no; 1: yes)	
NestSite_cliff	Nest Site: Cliff and bank	Whether using a nest built on cliff or banks	binary (0: no; 1: yes)	
NestSite_underground	Nest Site: Underground	Whether using a nest built underground	binary (0: no; 1: yes)	
NestSite_waterbody	Nest Site: Waterbody	Whether using a nest built in water bodies	binary (0: no; 1: yes)	
NestSite_termite	Nest Site: Termite/ant nest	Whether using a nest built in termite or ant nests	binary (0: no; 1: yes)	
NestAtt_basal	Nest attachment: Basal	Whether using a nest with basal attachment	binary (0: no; 1: yes)	
NestAtt_forked	Nest attachment: Horizontally forked	Whether using a nest with horizontally-forked attachment	binary (0: no; 1: yes)	
NestAtt_lateral	Nest attachment: Lateral	Whether using a nest with lateral attachment	binary (0: no; 1: yes)	
NestAtt_pensile	Nest attachment: Pensile	Whether using a nest with pensile attachment	binary (0: no; 1: yes)	

**Table 3.** Sources of the data (DataSource) for six morphological and four breeding traits (TraitName) of the bird species in the dataset. The number of measurements (SampleSize) used for calculating the trait values and the calculation method (Method) are also provided. The scientific name following the HBW and BirdLife Taxonomic Checklist Version 4.0 (ScientificName) and the taxonomic identifier used by BirdLife International (SISRecID) are provided for each species.

**SISRecID**	**ScientificName**	**TraitName**	**SampleSize**	Method^*^	**DataSource^#^**
22680107	*Aix galericulata*	BodyLength	NA	2	HBW
22680107	*Aix galericulata*	BodyMass	15	1	AoT
22680107	*Aix galericulata*	HeadLength	1	1	ASIZ
22680107	*Aix galericulata*	TailLength	18	1	AoT; TM
22680107	*Aix galericulata*	TarsusLength	16	1	AoT; ASIZ
22680107	*Aix galericulata*	WingLength	18	1	AoT; TM
22680107	*Aix galericulata*	ClutchSize	NA	2	AoT
22680107	*Aix galericulata*	IncuPeriod	NA	2	HBW
22680107	*Aix galericulata*	EggLength	NA	2	HBW
22680107	*Aix galericulata*	EggWidth	NA	2	HBW
22736042	*Anas zonorhyncha*	BodyLength	3	1	ASIZ
22736042	*Anas zonorhyncha*	BodyMass	1	1	ASIZ
22736042	*Anas zonorhyncha*	HeadLength	2	1	ASIZ
22736042	*Anas zonorhyncha*	TailLength	6	1	AoT; ASIZ
22736042	*Anas zonorhyncha*	TarsusLength	6	1	AoT; ASIZ
22736042	*Anas zonorhyncha*	WingLength	6	1	AoT; ASIZ
22736042	*Anas zonorhyncha*	ClutchSize	NA	2	AoT
22736042	*Anas zonorhyncha*	IncuPeriod	NA	2	AoT
22736042	*Anas zonorhyncha*	EggLength	NA	2	HBW
22736042	*Anas zonorhyncha*	EggWidth	NA	2	HBW
22678979	*Synoicus chinensis*	BodyLength	1	1	ASIZ
22678979	*Synoicus chinensis*	BodyMass	5	1	AoT; ASIZ
22678979	*Synoicus chinensis*	HeadLength	1	1	ASIZ
22678979	*Synoicus chinensis*	TailLength	5	1	AoT; ASIZ
22678979	*Synoicus chinensis*	TarsusLength	6	1	AoT; ASIZ
22678979	*Synoicus chinensis*	WingLength	6	1	AoT; ASIZ
22678979	*Synoicus chinensis*	ClutchSize	1	3	AoT
22678979	*Synoicus chinensis*	IncuPeriod	NA	2	HBW
22678979	*Synoicus chinensis*	EggLength	NA	2	HBW
22678979	*Synoicus chinensis*	EggWidth	NA	2	HBW
22679026	*Arborophila crudigularis*	BodyLength	2	1	ASIZ
22679026	*Arborophila crudigularis*	BodyMass	10	1	AoT; MAPS
22679026	*Arborophila crudigularis*	HeadLength	5	1	ASIZ; MAPS
22679026	*Arborophila crudigularis*	TailLength	32	1	AoT; ASIZ; MAPS; TM
22679026	*Arborophila crudigularis*	TarsusLength	16	1	AoT; ASIZ; MAPS
22679026	*Arborophila crudigularis*	WingLength	33	1	AoT; ASIZ; MAPS; TM
22679026	*Arborophila crudigularis*	ClutchSize	3	2	AoT
22679026	*Arborophila crudigularis*	IncuPeriod	NA	3	AoT
22679026	*Arborophila crudigularis*	EggLength	60	3	AoT
22679026	*Arborophila crudigularis*	EggWidth	60	3	AoT
22725205	*Bambusicola sonorivox*	BodyLength	10	1	ASIZ
22725205	*Bambusicola sonorivox*	BodyMass	21	1	AoT; ASIZ; MAPS
22725205	*Bambusicola sonorivox*	HeadLength	14	1	ASIZ; MAPS
22725205	*Bambusicola sonorivox*	TailLength	33	1	AoT; ASIZ; MAPS; TM
22725205	*Bambusicola sonorivox*	TarsusLength	25	1	AoT; ASIZ; MAPS
22725205	*Bambusicola sonorivox*	WingLength	35	1	AoT; ASIZ; MAPS; TM
22725205	*Bambusicola sonorivox*	ClutchSize	NA	2	AoT
22725205	*Bambusicola sonorivox*	IncuPeriod	NA	NA	NA
22725205	*Bambusicola sonorivox*	EggLength	NA	2	AoT
22725205	*Bambusicola sonorivox*	EggWidth	NA	2	AoT
22679336	*Syrmaticus mikado*	BodyLength	NA	2	HBW
22679336	*Syrmaticus mikado*	BodyMass	4	1	AoT
22679336	*Syrmaticus mikado*	HeadLength	NA	NA	NA
22679336	*Syrmaticus mikado*	TailLength	18	1	AoT; TM
22679336	*Syrmaticus mikado*	TarsusLength	4	1	AoT
22679336	*Syrmaticus mikado*	WingLength	18	1	AoT; TM
22679336	*Syrmaticus mikado*	ClutchSize	NA	2	AoT
22679336	*Syrmaticus mikado*	IncuPeriod	NA	3	AoT
22679336	*Syrmaticus mikado*	EggLength	NA	3	AoT
22679336	*Syrmaticus mikado*	EggWidth	NA	3	AoT
45100023	*Phasianus colchicus*	BodyLength	2	1	ASIZ
45100023	*Phasianus colchicus*	BodyMass	15	1	AoT
45100023	*Phasianus colchicus*	HeadLength	1	1	ASIZ
45100023	*Phasianus colchicus*	TailLength	29	1	AoT; ASIZ; TM
45100023	*Phasianus colchicus*	TarsusLength	20	1	AoT; ASIZ
45100023	*Phasianus colchicus*	WingLength	33	1	AoT; ASIZ; TM
45100023	*Phasianus colchicus*	ClutchSize	NA	2	AoT
45100023	*Phasianus colchicus*	IncuPeriod	NA	2	AoT
45100023	*Phasianus colchicus*	EggLength	1054	3	AoT
45100023	*Phasianus colchicus*	EggWidth	1054	3	AoT
22679241	*Lophura swinhoii*	BodyLength	1	1	ASIZ
22679241	*Lophura swinhoii*	BodyMass	5	1	AoT
22679241	*Lophura swinhoii*	HeadLength	2	1	ASIZ; MAPS
22679241	*Lophura swinhoii*	TailLength	19	1	AoT; ASIZ; MAPS; TM
22679241	*Lophura swinhoii*	TarsusLength	8	1	AoT; ASIZ; MAPS
22679241	*Lophura swinhoii*	WingLength	21	1	AoT; ASIZ; MAPS; TM
22679241	*Lophura swinhoii*	ClutchSize	10	3	AoT
22679241	*Lophura swinhoii*	IncuPeriod	NA	2	AoT
22679241	*Lophura swinhoii*	EggLength	NA	3	AoT
22679241	*Lophura swinhoii*	EggWidth	NA	3	AoT
22696545	*Tachybaptus ruficollis*	BodyLength	NA	2	NBW
22696545	*Tachybaptus ruficollis*	BodyMass	5	1	AoT
22696545	*Tachybaptus ruficollis*	HeadLength	NA	NA	NA
22696545	*Tachybaptus ruficollis*	TailLength	4	1	AoT
22696545	*Tachybaptus ruficollis*	TarsusLength	10	1	AoT
22696545	*Tachybaptus ruficollis*	WingLength	15	1	AoT; TM
22696545	*Tachybaptus ruficollis*	ClutchSize	NA	2	AoT
22696545	*Tachybaptus ruficollis*	IncuPeriod	NA	2	HBW
22696545	*Tachybaptus ruficollis*	EggLength	NA	3	AoT
22696545	*Tachybaptus ruficollis*	EggWidth	NA	3	AoT
22697303	*Ixobrychus sinensis*	BodyLength	4	1	ASIZ
22697303	*Ixobrychus sinensis*	BodyMass	4	1	ASIZ
22697303	*Ixobrychus sinensis*	HeadLength	6	1	ASIZ
22697303	*Ixobrychus sinensis*	TailLength	11	1	AoT; ASIZ; TM
22697303	*Ixobrychus sinensis*	TarsusLength	10	1	AoT; ASIZ
22697303	*Ixobrychus sinensis*	WingLength	11	1	AoT; ASIZ; TM
22697303	*Ixobrychus sinensis*	ClutchSize	NA	2	AoT
22697303	*Ixobrychus sinensis*	IncuPeriod	NA	3	AoT
22697303	*Ixobrychus sinensis*	EggLength	NA	2	AoT
22697303	*Ixobrychus sinensis*	EggWidth	NA	2	AoT
22697323	*Ixobrychus cinnamomeus*	BodyLength	2	1	ASIZ
22697323	*Ixobrychus cinnamomeus*	BodyMass	2	1	ASIZ
22697323	*Ixobrychus cinnamomeus*	HeadLength	4	1	ASIZ
22697323	*Ixobrychus cinnamomeus*	TailLength	16	1	AoT; ASIZ; TM
22697323	*Ixobrychus cinnamomeus*	TarsusLength	16	1	AoT; ASIZ; TM
22697323	*Ixobrychus cinnamomeus*	WingLength	16	1	AoT; ASIZ; TM
22697323	*Ixobrychus cinnamomeus*	ClutchSize	NA	2	AoT
22697323	*Ixobrychus cinnamomeus*	IncuPeriod	NA	2	AoT
22697323	*Ixobrychus cinnamomeus*	EggLength	4	3	AoT
22697323	*Ixobrychus cinnamomeus*	EggWidth	4	3	AoT
22697031	*Ardea purpurea*	BodyLength	NA	2	HBW
22697031	*Ardea purpurea*	BodyMass	1	1	AoT
22697031	*Ardea purpurea*	HeadLength	NA	NA	NA
22697031	*Ardea purpurea*	TailLength	6	1	AoT; TM
22697031	*Ardea purpurea*	TarsusLength	2	1	AoT
22697031	*Ardea purpurea*	WingLength	6	1	AoT; TM
22697031	*Ardea purpurea*	ClutchSize	NA	2	HBW
22697031	*Ardea purpurea*	IncuPeriod	NA	2	HBW
22697031	*Ardea purpurea*	EggLength	NA	2	HBW
22697031	*Ardea purpurea*	EggWidth	NA	2	HBW
22697043	*Ardea alba*	BodyLength	NA	2	HBW
22697043	*Ardea alba*	BodyMass	5	1	AoT
22697043	*Ardea alba*	HeadLength	NA	NA	NA
22697043	*Ardea alba*	TailLength	9	1	AoT
22697043	*Ardea alba*	TarsusLength	9	1	AoT
22697043	*Ardea alba*	WingLength	9	1	AoT
22697043	*Ardea alba*	ClutchSize	NA	2	HBW
22697043	*Ardea alba*	IncuPeriod	NA	2	HBW
22697043	*Ardea alba*	EggLength	NA	3	HBW
22697043	*Ardea alba*	EggWidth	NA	3	HBW
62774969	*Egretta garzetta*	BodyLength	9	1	ASIZ
62774969	*Egretta garzetta*	BodyMass	9	1	ASIZ
62774969	*Egretta garzetta*	HeadLength	14	1	ASIZ
62774969	*Egretta garzetta*	TailLength	21	1	AoT; ASIZ
62774969	*Egretta garzetta*	TarsusLength	22	1	AoT; ASIZ
62774969	*Egretta garzetta*	WingLength	22	1	AoT; ASIZ
62774969	*Egretta garzetta*	ClutchSize	NA	2	AoT
62774969	*Egretta garzetta*	IncuPeriod	NA	2	AoT
62774969	*Egretta garzetta*	EggLength	NA	3	AoT
62774969	*Egretta garzetta*	EggWidth	NA	3	AoT
22696980	*Egretta sacra*	BodyLength	NA	2	HBW
22696980	*Egretta sacra*	BodyMass	NA	2	HBW
22696980	*Egretta sacra*	HeadLength	NA	NA	NA
22696980	*Egretta sacra*	TailLength	7	1	TM; YIO
22696980	*Egretta sacra*	TarsusLength	3	1	YIO
22696980	*Egretta sacra*	WingLength	7	1	TM; YIO
22696980	*Egretta sacra*	ClutchSize	NA	2	AoT
22696980	*Egretta sacra*	IncuPeriod	NA	2	AoT
22696980	*Egretta sacra*	EggLength	20	3	AoT
22696980	*Egretta sacra*	EggWidth	20	3	AoT
22697109	*Bubulcus ibis*	BodyLength	14	1	ASIZ
22697109	*Bubulcus ibis*	BodyMass	9	1	ASIZ
22697109	*Bubulcus ibis*	HeadLength	17	1	ASIZ
22697109	*Bubulcus ibis*	TailLength	32	1	AoT; ASIZ
22697109	*Bubulcus ibis*	TarsusLength	32	1	AoT; ASIZ
22697109	*Bubulcus ibis*	WingLength	32	1	AoT; ASIZ
22697109	*Bubulcus ibis*	ClutchSize	NA	2	AoT
22697109	*Bubulcus ibis*	IncuPeriod	NA	2	AoT
22697109	*Bubulcus ibis*	EggLength	NA	2	HBW
22697109	*Bubulcus ibis*	EggWidth	NA	2	HBW
22728182	*Butorides striata*	BodyLength	1	1	ASIZ
22728182	*Butorides striata*	BodyMass	1	1	ASIZ
22728182	*Butorides striata*	HeadLength	1	1	ASIZ
22728182	*Butorides striata*	TailLength	6	1	AoT; ASIZ; TM
22728182	*Butorides striata*	TarsusLength	2	1	AoT; ASIZ
22728182	*Butorides striata*	WingLength	6	1	AoT; ASIZ; TM
22728182	*Butorides striata*	ClutchSize	NA	2	AoT
22728182	*Butorides striata*	IncuPeriod	NA	2	AoT
22728182	*Butorides striata*	EggLength	NA	3	HBW
22728182	*Butorides striata*	EggWidth	NA	3	HBW
22697211	*Nycticorax nycticorax*	BodyLength	14	1	ASIZ
22697211	*Nycticorax nycticorax*	BodyMass	12	1	ASIZ
22697211	*Nycticorax nycticorax*	HeadLength	23	1	ASIZ
22697211	*Nycticorax nycticorax*	TailLength	40	1	AoT; ASIZ; MAPS
22697211	*Nycticorax nycticorax*	TarsusLength	41	1	AoT; ASIZ; MAPS
22697211	*Nycticorax nycticorax*	WingLength	41	1	AoT; ASIZ; MAPS
22697211	*Nycticorax nycticorax*	ClutchSize	NA	2	AoT
22697211	*Nycticorax nycticorax*	IncuPeriod	NA	2	AoT
22697211	*Nycticorax nycticorax*	EggLength	NA	3	AoT
22697211	*Nycticorax nycticorax*	EggWidth	NA	3	AoT
22697242	*Gorsachius melanolophus*	BodyLength	13	1	ASIZ
22697242	*Gorsachius melanolophus*	BodyMass	5	1	ASIZ; MAPS
22697242	*Gorsachius melanolophus*	HeadLength	22	1	ASIZ; MAPS
22697242	*Gorsachius melanolophus*	TailLength	24	1	AoT; ASIZ; MAPS
22697242	*Gorsachius melanolophus*	TarsusLength	25	1	AoT; ASIZ; MAPS
22697242	*Gorsachius melanolophus*	WingLength	23	1	AoT; ASIZ; MAPS
22697242	*Gorsachius melanolophus*	ClutchSize	NA	2	AoT
22697242	*Gorsachius melanolophus*	IncuPeriod	NA	2	AoT
22697242	*Gorsachius melanolophus*	EggLength	1	3	HBW
22697242	*Gorsachius melanolophus*	EggWidth	1	3	HBW
22695028	*Elanus caeruleus*	BodyLength	1	1	ASIZ
22695028	*Elanus caeruleus*	BodyMass	NA	2	HBW
22695028	*Elanus caeruleus*	HeadLength	1	1	ASIZ
22695028	*Elanus caeruleus*	TailLength	2	1	AoT; ASIZ
22695028	*Elanus caeruleus*	TarsusLength	3	1	AoT; ASIZ
22695028	*Elanus caeruleus*	WingLength	3	1	AoT; ASIZ
22695028	*Elanus caeruleus*	ClutchSize	NA	2	AoT
22695028	*Elanus caeruleus*	IncuPeriod	NA	2	HBW
22695028	*Elanus caeruleus*	EggLength	NA	3	HBW
22695028	*Elanus caeruleus*	EggWidth	NA	3	HBW
22694995	*Pernis ptilorhynchus*	BodyLength	NA	2	HBW
22694995	*Pernis ptilorhynchus*	BodyMass	8	1	AoT
22694995	*Pernis ptilorhynchus*	HeadLength	1	1	ASIZ
22694995	*Pernis ptilorhynchus*	TailLength	8	1	AoT
22694995	*Pernis ptilorhynchus*	TarsusLength	9	1	AoT; ASIZ
22694995	*Pernis ptilorhynchus*	WingLength	8	1	AoT
22694995	*Pernis ptilorhynchus*	ClutchSize	NA	3	AoT
22694995	*Pernis ptilorhynchus*	IncuPeriod	NA	2	HBW
22694995	*Pernis ptilorhynchus*	EggLength	NA	NA	NA
22694995	*Pernis ptilorhynchus*	EggWidth	NA	NA	NA
22695293	*Spilornis cheela*	BodyLength	17	1	ASIZ
22695293	*Spilornis cheela*	BodyMass	6	1	ASIZ
22695293	*Spilornis cheela*	HeadLength	20	1	ASIZ
22695293	*Spilornis cheela*	TailLength	31	1	AoT; ASIZ
22695293	*Spilornis cheela*	TarsusLength	31	1	AoT; ASIZ
22695293	*Spilornis cheela*	WingLength	31	1	AoT; ASIZ
22695293	*Spilornis cheela*	ClutchSize	NA	3	AoT
22695293	*Spilornis cheela*	IncuPeriod	NA	2	HBW
22695293	*Spilornis cheela*	EggLength	1	3	AoT
22695293	*Spilornis cheela*	EggWidth	1	3	AoT
22696153	*Nisaetus nipalensis*	BodyLength	NA	2	HBW
22696153	*Nisaetus nipalensis*	BodyMass	3	1	AoT
22696153	*Nisaetus nipalensis*	HeadLength	1	1	ASIZ
22696153	*Nisaetus nipalensis*	TailLength	4	1	AoT; ASIZ
22696153	*Nisaetus nipalensis*	TarsusLength	4	1	AoT; ASIZ
22696153	*Nisaetus nipalensis*	WingLength	4	1	AoT; ASIZ
22696153	*Nisaetus nipalensis*	ClutchSize	NA	3	AoT
22696153	*Nisaetus nipalensis*	IncuPeriod	NA	2	HBW
22696153	*Nisaetus nipalensis*	EggLength	NA	3	HBW
22696153	*Nisaetus nipalensis*	EggWidth	NA	3	HBW
22696019	*Ictinaetus malaiensis*	BodyLength	1	1	YIO
22696019	*Ictinaetus malaiensis*	BodyMass	NA	2	HBW
22696019	*Ictinaetus malaiensis*	HeadLength	1	1	YIO
22696019	*Ictinaetus malaiensis*	TailLength	1	1	YIO
22696019	*Ictinaetus malaiensis*	TarsusLength	1	1	AoT
22696019	*Ictinaetus malaiensis*	WingLength	2	1	AoT; YIO
22696019	*Ictinaetus malaiensis*	ClutchSize	NA	2	AoT
22696019	*Ictinaetus malaiensis*	IncuPeriod	NA	2	HBW
22696019	*Ictinaetus malaiensis*	EggLength	NA	NA	NA
22696019	*Ictinaetus malaiensis*	EggWidth	NA	NA	NA
22695462	*Accipiter trivirgatus*	BodyLength	19	1	ASIZ
22695462	*Accipiter trivirgatus*	BodyMass	9	1	ASIZ; MAPS
22695462	*Accipiter trivirgatus*	HeadLength	24	1	ASIZ; MAPS
22695462	*Accipiter trivirgatus*	TailLength	37	1	AoT; ASIZ; MAPS
22695462	*Accipiter trivirgatus*	TarsusLength	39	1	AoT; ASIZ; MAPS
22695462	*Accipiter trivirgatus*	WingLength	36	1	AoT; ASIZ; MAPS
22695462	*Accipiter trivirgatus*	ClutchSize	NA	3	AoT
22695462	*Accipiter trivirgatus*	IncuPeriod	NA	3	AoT
22695462	*Accipiter trivirgatus*	EggLength	NA	3	AoT
22695462	*Accipiter trivirgatus*	EggWidth	NA	3	AoT
22695588	*Accipiter virgatus*	BodyLength	8	1	ASIZ
22695588	*Accipiter virgatus*	BodyMass	1	1	ASIZ
22695588	*Accipiter virgatus*	HeadLength	9	1	ASIZ; MAPS
22695588	*Accipiter virgatus*	TailLength	23	1	AoT; ASIZ; MAPS
22695588	*Accipiter virgatus*	TarsusLength	21	1	AoT; ASIZ; MAPS
22695588	*Accipiter virgatus*	WingLength	22	1	AoT; ASIZ; MAPS
22695588	*Accipiter virgatus*	ClutchSize	NA	2	AoT
22695588	*Accipiter virgatus*	IncuPeriod	NA	2	AoT
22695588	*Accipiter virgatus*	EggLength	4	3	AoT
22695588	*Accipiter virgatus*	EggWidth	4	3	AoT
22734972	*Milvus migrans*	BodyLength	1	1	ASIZ
22734972	*Milvus migrans*	BodyMass	NA	2	HBW
22734972	*Milvus migrans*	HeadLength	1	1	ASIZ
22734972	*Milvus migrans*	TailLength	5	1	AoT; ASIZ
22734972	*Milvus migrans*	TarsusLength	5	1	AoT; ASIZ
22734972	*Milvus migrans*	WingLength	5	1	AoT; ASIZ
22734972	*Milvus migrans*	ClutchSize	NA	2	AoT
22734972	*Milvus migrans*	IncuPeriod	NA	2	AoT
22734972	*Milvus migrans*	EggLength	NA	3	AoT
22734972	*Milvus migrans*	EggWidth	NA	3	AoT
22692317	*Rallina eurizonoides*	BodyLength	7	1	ASIZ
22692317	*Rallina eurizonoides*	BodyMass	15	1	AoT; ASIZ
22692317	*Rallina eurizonoides*	HeadLength	6	1	ASIZ
22692317	*Rallina eurizonoides*	TailLength	16	1	AoT; ASIZ
22692317	*Rallina eurizonoides*	TarsusLength	17	1	AoT; ASIZ
22692317	*Rallina eurizonoides*	WingLength	17	1	AoT; ASIZ
22692317	*Rallina eurizonoides*	ClutchSize	NA	2	HBW
22692317	*Rallina eurizonoides*	IncuPeriod	NA	NA	NA
22692317	*Rallina eurizonoides*	EggLength	NA	NA	NA
22692317	*Rallina eurizonoides*	EggWidth	NA	NA	NA
22692463	*Lewinia striata*	BodyLength	2	1	ASIZ
22692463	*Lewinia striata*	BodyMass	4	1	AoT
22692463	*Lewinia striata*	HeadLength	2	1	ASIZ
22692463	*Lewinia striata*	TailLength	5	1	AoT; ASIZ
22692463	*Lewinia striata*	TarsusLength	7	1	AoT; ASIZ
22692463	*Lewinia striata*	WingLength	7	1	AoT; ASIZ
22692463	*Lewinia striata*	ClutchSize	1	3	AoT
22692463	*Lewinia striata*	IncuPeriod	NA	2	HBW
22692463	*Lewinia striata*	EggLength	NA	3	AoT
22692463	*Lewinia striata*	EggWidth	NA	3	AoT
22692640	*Amaurornis phoenicurus*	BodyLength	5	1	ASIZ
22692640	*Amaurornis phoenicurus*	BodyMass	11	1	AoT; ASIZ
22692640	*Amaurornis phoenicurus*	HeadLength	8	1	ASIZ
22692640	*Amaurornis phoenicurus*	TailLength	15	1	AoT; ASIZ
22692640	*Amaurornis phoenicurus*	TarsusLength	17	1	AoT; ASIZ
22692640	*Amaurornis phoenicurus*	WingLength	17	1	AoT; ASIZ
22692640	*Amaurornis phoenicurus*	ClutchSize	NA	2	AoT
22692640	*Amaurornis phoenicurus*	IncuPeriod	NA	3	HBW
22692640	*Amaurornis phoenicurus*	EggLength	NA	3	AoT
22692640	*Amaurornis phoenicurus*	EggWidth	NA	3	AoT
22692699	*Zapornia fusca*	BodyLength	2	1	ASIZ
22692699	*Zapornia fusca*	BodyMass	13	1	AoT; ASIZ
22692699	*Zapornia fusca*	HeadLength	2	1	ASIZ
22692699	*Zapornia fusca*	TailLength	13	1	AoT; ASIZ
22692699	*Zapornia fusca*	TarsusLength	14	1	AoT; ASIZ
22692699	*Zapornia fusca*	WingLength	14	1	AoT; ASIZ
22692699	*Zapornia fusca*	ClutchSize	NA	2	HBW
22692699	*Zapornia fusca*	IncuPeriod	NA	3	AoT
22692699	*Zapornia fusca*	EggLength	NA	3	AoT
22692699	*Zapornia fusca*	EggWidth	NA	3	AoT
22692789	*Gallicrex cinerea*	BodyLength	4	1	ASIZ
22692789	*Gallicrex cinerea*	BodyMass	7	1	AoT; ASIZ
22692789	*Gallicrex cinerea*	HeadLength	4	1	ASIZ
22692789	*Gallicrex cinerea*	TailLength	11	1	AoT; ASIZ
22692789	*Gallicrex cinerea*	TarsusLength	12	1	AoT; ASIZ
22692789	*Gallicrex cinerea*	WingLength	12	1	AoT; ASIZ
22692789	*Gallicrex cinerea*	ClutchSize	NA	3	AoT
22692789	*Gallicrex cinerea*	IncuPeriod	NA	NA	NA
22692789	*Gallicrex cinerea*	EggLength	NA	NA	NA
22692789	*Gallicrex cinerea*	EggWidth	NA	NA	NA
62120190	*Gallinula chloropus*	BodyLength	5	1	ASIZ
62120190	*Gallinula chloropus*	BodyMass	9	1	AoT; ASIZ
62120190	*Gallinula chloropus*	HeadLength	7	1	ASIZ
62120190	*Gallinula chloropus*	TailLength	13	1	AoT; ASIZ
62120190	*Gallinula chloropus*	TarsusLength	14	1	AoT; ASIZ
62120190	*Gallinula chloropus*	WingLength	13	1	AoT; ASIZ
62120190	*Gallinula chloropus*	ClutchSize	NA	2	AoT
62120190	*Gallinula chloropus*	IncuPeriod	NA	2	AoT
62120190	*Gallinula chloropus*	EggLength	NA	3	AoT
62120190	*Gallinula chloropus*	EggWidth	NA	3	AoT
22727969	*Himantopus himantopus*	BodyLength	1	1	ASIZ
22727969	*Himantopus himantopus*	BodyMass	1	1	ASIZ
22727969	*Himantopus himantopus*	HeadLength	3	1	ASIZ
22727969	*Himantopus himantopus*	TailLength	3	1	ASIZ
22727969	*Himantopus himantopus*	TarsusLength	3	1	ASIZ
22727969	*Himantopus himantopus*	WingLength	3	1	ASIZ
22727969	*Himantopus himantopus*	ClutchSize	NA	3	AoT
22727969	*Himantopus himantopus*	IncuPeriod	NA	2	AoT
22727969	*Himantopus himantopus*	EggLength	NA	2	HBW
22727969	*Himantopus himantopus*	EggWidth	NA	2	HBW
22727487	*Charadrius alexandrinus*	BodyLength	6	1	ASIZ
22727487	*Charadrius alexandrinus*	BodyMass	2	1	ASIZ
22727487	*Charadrius alexandrinus*	HeadLength	8	1	ASIZ
22727487	*Charadrius alexandrinus*	TailLength	27	1	AoT; ASIZ
22727487	*Charadrius alexandrinus*	TarsusLength	27	1	AoT; ASIZ
22727487	*Charadrius alexandrinus*	WingLength	27	1	AoT; ASIZ
22727487	*Charadrius alexandrinus*	ClutchSize	NA	2	AoT
22727487	*Charadrius alexandrinus*	IncuPeriod	NA	2	AoT
22727487	*Charadrius alexandrinus*	EggLength	NA	3	AoT
22727487	*Charadrius alexandrinus*	EggWidth	NA	3	AoT
22693770	*Charadrius dubius*	BodyLength	3	1	ASIZ
22693770	*Charadrius dubius*	BodyMass	2	1	ASIZ
22693770	*Charadrius dubius*	HeadLength	3	1	ASIZ
22693770	*Charadrius dubius*	TailLength	12	1	AoT; ASIZ
22693770	*Charadrius dubius*	TarsusLength	12	1	AoT; ASIZ
22693770	*Charadrius dubius*	WingLength	12	1	AoT; ASIZ
22693770	*Charadrius dubius*	ClutchSize	NA	2	AoT
22693770	*Charadrius dubius*	IncuPeriod	NA	2	HBW
22693770	*Charadrius dubius*	EggLength	NA	3	HBW
22693770	*Charadrius dubius*	EggWidth	NA	3	HBW
22735810	*Rostratula benghalensis*	BodyLength	18	1	ASIZ
22735810	*Rostratula benghalensis*	BodyMass	12	1	AoT; ASIZ
22735810	*Rostratula benghalensis*	HeadLength	21	1	ASIZ
22735810	*Rostratula benghalensis*	TailLength	22	1	AoT; ASIZ
22735810	*Rostratula benghalensis*	TarsusLength	23	1	AoT; ASIZ
22735810	*Rostratula benghalensis*	WingLength	22	1	AoT; ASIZ
22735810	*Rostratula benghalensis*	ClutchSize	NA	2	AoT
22735810	*Rostratula benghalensis*	IncuPeriod	NA	2	AoT
22735810	*Rostratula benghalensis*	EggLength	NA	2	HBW
22735810	*Rostratula benghalensis*	EggWidth	NA	2	HBW
22693543	*Hydrophasianus chirurgus*	BodyLength	10	1	ASIZ
22693543	*Hydrophasianus chirurgus*	BodyMass	3	1	AoT
22693543	*Hydrophasianus chirurgus*	HeadLength	11	1	ASIZ
22693543	*Hydrophasianus chirurgus*	TailLength	14	1	AoT; ASIZ
22693543	*Hydrophasianus chirurgus*	TarsusLength	15	1	AoT; ASIZ
22693543	*Hydrophasianus chirurgus*	WingLength	15	1	AoT; ASIZ
22693543	*Hydrophasianus chirurgus*	ClutchSize	NA	3	AoT
22693543	*Hydrophasianus chirurgus*	IncuPeriod	NA	2	AoT
22693543	*Hydrophasianus chirurgus*	EggLength	16	3	AoT
22693543	*Hydrophasianus chirurgus*	EggWidth	16	3	AoT
22680500	*Turnix sylvaticus*	BodyLength	1	1	YIO
22680500	*Turnix sylvaticus*	BodyMass	NA	2	HBW
22680500	*Turnix sylvaticus*	HeadLength	1	1	YIO
22680500	*Turnix sylvaticus*	TailLength	2	1	AoT; YIO
22680500	*Turnix sylvaticus*	TarsusLength	2	1	AoT; YIO
22680500	*Turnix sylvaticus*	WingLength	2	1	AoT; YIO
22680500	*Turnix sylvaticus*	ClutchSize	NA	2	AoT
22680500	*Turnix sylvaticus*	IncuPeriod	NA	2	HBW
22680500	*Turnix sylvaticus*	EggLength	NA	3	AoT
22680500	*Turnix sylvaticus*	EggWidth	NA	3	AoT
22680549	*Turnix suscitator*	BodyLength	4	1	ASIZ
22680549	*Turnix suscitator*	BodyMass	2	1	ASIZ
22680549	*Turnix suscitator*	HeadLength	5	1	ASIZ
22680549	*Turnix suscitator*	TailLength	14	1	AoT; ASIZ
22680549	*Turnix suscitator*	TarsusLength	15	1	AoT; ASIZ
22680549	*Turnix suscitator*	WingLength	15	1	AoT; ASIZ
22680549	*Turnix suscitator*	ClutchSize	NA	3	AoT
22680549	*Turnix suscitator*	IncuPeriod	NA	2	HBW
22680549	*Turnix suscitator*	EggLength	NA	3	AoT
22680549	*Turnix suscitator*	EggWidth	NA	3	AoT
22694132	*Glareola maldivarum*	BodyLength	10	1	ASIZ
22694132	*Glareola maldivarum*	BodyMass	10	1	AoT; ASIZ
22694132	*Glareola maldivarum*	HeadLength	10	1	ASIZ
22694132	*Glareola maldivarum*	TailLength	12	1	AoT; ASIZ
22694132	*Glareola maldivarum*	TarsusLength	10	1	ASIZ
22694132	*Glareola maldivarum*	WingLength	12	1	AoT; ASIZ
22694132	*Glareola maldivarum*	ClutchSize	NA	2	AoT
22694132	*Glareola maldivarum*	IncuPeriod	7	3	AoT
22694132	*Glareola maldivarum*	EggLength	NA	NA	NA
22694132	*Glareola maldivarum*	EggWidth	NA	NA	NA
22694794	*Anous stolidus*	BodyLength	3	1	ASIZ
22694794	*Anous stolidus*	BodyMass	1	1	AoT
22694794	*Anous stolidus*	HeadLength	3	1	ASIZ
22694794	*Anous stolidus*	TailLength	5	1	AoT; ASIZ
22694794	*Anous stolidus*	TarsusLength	5	1	AoT; ASIZ
22694794	*Anous stolidus*	WingLength	5	1	AoT; ASIZ
22694794	*Anous stolidus*	ClutchSize	NA	3	AoT
22694794	*Anous stolidus*	IncuPeriod	NA	2	AoT
22694794	*Anous stolidus*	EggLength	NA	NA	NA
22694794	*Anous stolidus*	EggWidth	NA	NA	NA
22694740	*Onychoprion fuscatus*	BodyLength	3	1	ASIZ
22694740	*Onychoprion fuscatus*	BodyMass	NA	2	HBW
22694740	*Onychoprion fuscatus*	HeadLength	5	1	ASIZ
22694740	*Onychoprion fuscatus*	TailLength	10	1	AoT; ASIZ
22694740	*Onychoprion fuscatus*	TarsusLength	13	1	AoT; ASIZ
22694740	*Onychoprion fuscatus*	WingLength	11	1	AoT; ASIZ
22694740	*Onychoprion fuscatus*	ClutchSize	NA	2	HBW
22694740	*Onychoprion fuscatus*	IncuPeriod	NA	2	HBW
22694740	*Onychoprion fuscatus*	EggLength	NA	NA	NA
22694740	*Onychoprion fuscatus*	EggWidth	NA	NA	NA
22694730	*Onychoprion anaethetus*	BodyLength	10	1	YIO
22694730	*Onychoprion anaethetus*	BodyMass	NA	2	HBW
22694730	*Onychoprion anaethetus*	HeadLength	10	1	YIO
22694730	*Onychoprion anaethetus*	TailLength	12	1	AoT; YIO
22694730	*Onychoprion anaethetus*	TarsusLength	12	1	AoT; YIO
22694730	*Onychoprion anaethetus*	WingLength	12	1	AoT; YIO
22694730	*Onychoprion anaethetus*	ClutchSize	NA	2	AoT
22694730	*Onychoprion anaethetus*	IncuPeriod	NA	2	AoT
22694730	*Onychoprion anaethetus*	EggLength	NA	NA	NA
22694730	*Onychoprion anaethetus*	EggWidth	NA	NA	NA
22694656	*Sternula albifrons*	BodyLength	5	1	ASIZ
22694656	*Sternula albifrons*	BodyMass	3	1	ASIZ
22694656	*Sternula albifrons*	HeadLength	7	1	ASIZ
22694656	*Sternula albifrons*	TailLength	8	1	ASIZ
22694656	*Sternula albifrons*	TarsusLength	14	1	AoT; ASIZ
22694656	*Sternula albifrons*	WingLength	11	1	AoT; ASIZ
22694656	*Sternula albifrons*	ClutchSize	NA	2	AoT
22694656	*Sternula albifrons*	IncuPeriod	NA	3	AoT
22694656	*Sternula albifrons*	EggLength	NA	NA	NA
22694656	*Sternula albifrons*	EggWidth	NA	NA	NA
22694601	*Sterna dougallii*	BodyLength	NA	2	HBW
22694601	*Sterna dougallii*	BodyMass	13	1	AoT
22694601	*Sterna dougallii*	HeadLength	NA	NA	NA
22694601	*Sterna dougallii*	TailLength	1	1	AoT
22694601	*Sterna dougallii*	TarsusLength	14	1	AoT
22694601	*Sterna dougallii*	WingLength	14	1	AoT
22694601	*Sterna dougallii*	ClutchSize	NA	2	AoT
22694601	*Sterna dougallii*	IncuPeriod	NA	2	AoT
22694601	*Sterna dougallii*	EggLength	NA	NA	NA
22694601	*Sterna dougallii*	EggWidth	NA	NA	NA
22694612	*Sterna sumatrana*	BodyLength	NA	2	HBW
22694612	*Sterna sumatrana*	BodyMass	11	1	AoT
22694612	*Sterna sumatrana*	HeadLength	NA	NA	NA
22694612	*Sterna sumatrana*	TailLength	NA	NA	NA
22694612	*Sterna sumatrana*	TarsusLength	11	1	AoT
22694612	*Sterna sumatrana*	WingLength	11	1	AoT
22694612	*Sterna sumatrana*	ClutchSize	NA	2	AoT
22694612	*Sterna sumatrana*	IncuPeriod	NA	3	AoT
22694612	*Sterna sumatrana*	EggLength	NA	NA	NA
22694612	*Sterna sumatrana*	EggWidth	NA	NA	NA
22694571	*Thalasseus bergii*	BodyLength	NA	2	HBW
22694571	*Thalasseus bergii*	BodyMass	NA	2	HBW
22694571	*Thalasseus bergii*	HeadLength	1	1	ASIZ
22694571	*Thalasseus bergii*	TailLength	1	1	AoT
22694571	*Thalasseus bergii*	TarsusLength	2	1	AoT; ASIZ
22694571	*Thalasseus bergii*	WingLength	1	1	AoT
22694571	*Thalasseus bergii*	ClutchSize	NA	2	AoT
22694571	*Thalasseus bergii*	IncuPeriod	NA	2	AoT
22694571	*Thalasseus bergii*	EggLength	NA	NA	NA
22694571	*Thalasseus bergii*	EggWidth	NA	NA	NA
22694585	*Thalasseus bernsteini*	BodyLength	NA	2	HBW
22694585	*Thalasseus bernsteini*	BodyMass	NA	2	HBW
22694585	*Thalasseus bernsteini*	HeadLength	NA	NA	NA
22694585	*Thalasseus bernsteini*	TailLength	NA	NA	NA
22694585	*Thalasseus bernsteini*	TarsusLength	NA	NA	NA
22694585	*Thalasseus bernsteini*	WingLength	NA	NA	NA
22694585	*Thalasseus bernsteini*	ClutchSize	NA	3	AoT
22694585	*Thalasseus bernsteini*	IncuPeriod	NA	2	HBW
22694585	*Thalasseus bernsteini*	EggLength	NA	NA	NA
22694585	*Thalasseus bernsteini*	EggWidth	NA	NA	NA
22690168	*Columba pulchricollis*	BodyLength	1	1	ASIZ
22690168	*Columba pulchricollis*	BodyMass	NA	3	HBW
22690168	*Columba pulchricollis*	HeadLength	1	1	ASIZ
22690168	*Columba pulchricollis*	TailLength	9	1	AoT; ASIZ
22690168	*Columba pulchricollis*	TarsusLength	9	1	AoT; ASIZ
22690168	*Columba pulchricollis*	WingLength	9	1	AoT; ASIZ
22690168	*Columba pulchricollis*	ClutchSize	NA	2	AoT
22690168	*Columba pulchricollis*	IncuPeriod	NA	2	AoT
22690168	*Columba pulchricollis*	EggLength	NA	NA	NA
22690168	*Columba pulchricollis*	EggWidth	NA	NA	NA
22690439	*Streptopelia orientalis*	BodyLength	10	1	ASIZ
22690439	*Streptopelia orientalis*	BodyMass	6	1	ASIZ
22690439	*Streptopelia orientalis*	HeadLength	11	1	ASIZ
22690439	*Streptopelia orientalis*	TailLength	15	1	AoT; ASIZ
22690439	*Streptopelia orientalis*	TarsusLength	16	1	AoT; ASIZ
22690439	*Streptopelia orientalis*	WingLength	16	1	AoT; ASIZ
22690439	*Streptopelia orientalis*	ClutchSize	NA	3	AoT; ASIZ
22690439	*Streptopelia orientalis*	IncuPeriod	NA	2	AoT; ASIZ
22690439	*Streptopelia orientalis*	EggLength	NA	NA	NA
22690439	*Streptopelia orientalis*	EggWidth	NA	NA	NA
22690493	*Streptopelia tranquebarica*	BodyLength	21	1	ASIZ
22690493	*Streptopelia tranquebarica*	BodyMass	20	1	ASIZ; MAPS
22690493	*Streptopelia tranquebarica*	HeadLength	26	1	ASIZ; MAPS
22690493	*Streptopelia tranquebarica*	TailLength	35	1	AoT; ASIZ; MAPS
22690493	*Streptopelia tranquebarica*	TarsusLength	36	1	AoT; ASIZ; MAPS
22690493	*Streptopelia tranquebarica*	WingLength	36	1	AoT; ASIZ; MAPS
22690493	*Streptopelia tranquebarica*	ClutchSize	NA	3	AoT
22690493	*Streptopelia tranquebarica*	IncuPeriod	NA	NA	NA
22690493	*Streptopelia tranquebarica*	EggLength	NA	3	AoT
22690493	*Streptopelia tranquebarica*	EggWidth	NA	3	AoT
60482887	*Spilopelia chinensis*	BodyLength	17	1	ASIZ
60482887	*Spilopelia chinensis*	BodyMass	19	1	ASIZ; MAPS
60482887	*Spilopelia chinensis*	HeadLength	25	1	ASIZ; MAPS
60482887	*Spilopelia chinensis*	TailLength	27	1	AoT; ASIZ; MAPS
60482887	*Spilopelia chinensis*	TarsusLength	27	1	AoT; ASIZ; MAPS
60482887	*Spilopelia chinensis*	WingLength	29	1	AoT; ASIZ; MAPS
60482887	*Spilopelia chinensis*	ClutchSize	NA	3	AoT
60482887	*Spilopelia chinensis*	IncuPeriod	NA	2	HBW
60482887	*Spilopelia chinensis*	EggLength	NA	3	AoT
60482887	*Spilopelia chinensis*	EggWidth	NA	3	AoT
22690553	*Macropygia tenuirostris*	BodyLength	1	1	ASIZ
22690553	*Macropygia tenuirostris*	BodyMass	NA	2	HBW
22690553	*Macropygia tenuirostris*	HeadLength	1	1	ASIZ
22690553	*Macropygia tenuirostris*	TailLength	2	1	AoT; ASIZ
22690553	*Macropygia tenuirostris*	TarsusLength	2	1	AoT; ASIZ
22690553	*Macropygia tenuirostris*	WingLength	2	1	AoT; ASIZ
22690553	*Macropygia tenuirostris*	ClutchSize	NA	3	HBW
22690553	*Macropygia tenuirostris*	IncuPeriod	NA	NA	NA
22690553	*Macropygia tenuirostris*	EggLength	NA	3	AoT
22690553	*Macropygia tenuirostris*	EggWidth	NA	3	AoT
22725538	*Chalcophaps indica*	BodyLength	22	1	ASIZ
22725538	*Chalcophaps indica*	BodyMass	79	1	ASIZ; MAPS
22725538	*Chalcophaps indica*	HeadLength	91	1	ASIZ; MAPS
22725538	*Chalcophaps indica*	TailLength	99	1	AoT; ASIZ; MAPS
22725538	*Chalcophaps indica*	TarsusLength	103	1	AoT; ASIZ; MAPS
22725538	*Chalcophaps indica*	WingLength	104	1	AoT; ASIZ; MAPS
22725538	*Chalcophaps indica*	ClutchSize	NA	3	AoT
22725538	*Chalcophaps indica*	IncuPeriod	NA	2	AoT
22725538	*Chalcophaps indica*	EggLength	NA	NA	NA
22725538	*Chalcophaps indica*	EggWidth	NA	NA	NA
22691283	*Treron sieboldii*	BodyLength	12	1	ASIZ
22691283	*Treron sieboldii*	BodyMass	6	1	ASIZ
22691283	*Treron sieboldii*	HeadLength	10	1	ASIZ
22691283	*Treron sieboldii*	TailLength	21	1	AoT; ASIZ
22691283	*Treron sieboldii*	TarsusLength	22	1	AoT; ASIZ
22691283	*Treron sieboldii*	WingLength	22	1	AoT; ASIZ
22691283	*Treron sieboldii*	ClutchSize	NA	3	AoT
22691283	*Treron sieboldii*	IncuPeriod	NA	3	AoT
22691283	*Treron sieboldii*	EggLength	NA	3	AoT
22691283	*Treron sieboldii*	EggWidth	NA	3	AoT
22727539	*Treron formosae*	BodyLength	1	1	ASIZ
22727539	*Treron formosae*	BodyMass	1	1	ASIZ
22727539	*Treron formosae*	HeadLength	1	1	ASIZ
22727539	*Treron formosae*	TailLength	5	1	AoT; ASIZ
22727539	*Treron formosae*	TarsusLength	5	1	AoT; ASIZ
22727539	*Treron formosae*	WingLength	5	1	AoT; ASIZ
22727539	*Treron formosae*	ClutchSize	NA	3	AoT
22727539	*Treron formosae*	IncuPeriod	NA	NA	NA
22727539	*Treron formosae*	EggLength	NA	NA	NA
22727539	*Treron formosae*	EggWidth	NA	NA	NA
22691346	*Ramphiculus leclancheri*	BodyLength	NA	2	HBW
22691346	*Ramphiculus leclancheri*	BodyMass	NA	2	HBW
22691346	*Ramphiculus leclancheri*	HeadLength	NA	NA	NA
22691346	*Ramphiculus leclancheri*	TailLength	NA	NA	NA
22691346	*Ramphiculus leclancheri*	TarsusLength	NA	NA	NA
22691346	*Ramphiculus leclancheri*	WingLength	NA	NA	NA
22691346	*Ramphiculus leclancheri*	ClutchSize	NA	3	AoT
22691346	*Ramphiculus leclancheri*	IncuPeriod	NA	NA	NA
22691346	*Ramphiculus leclancheri*	EggLength	NA	NA	NA
22691346	*Ramphiculus leclancheri*	EggWidth	NA	NA	NA
22684254	*Centropus bengalensis*	BodyLength	11	1	ASIZ
22684254	*Centropus bengalensis*	BodyMass	11	1	ASIZ; MAPS
22684254	*Centropus bengalensis*	HeadLength	12	1	ASIZ; MAPS
22684254	*Centropus bengalensis*	TailLength	28	1	AoT; ASIZ; MAPS
22684254	*Centropus bengalensis*	TarsusLength	29	1	AoT; ASIZ; MAPS
22684254	*Centropus bengalensis*	WingLength	29	1	AoT; ASIZ; MAPS
22684254	*Centropus bengalensis*	ClutchSize	NA	2	AoT
22684254	*Centropus bengalensis*	IncuPeriod	NA	NA	NA
22684254	*Centropus bengalensis*	EggLength	NA	3	AoT
22684254	*Centropus bengalensis*	EggWidth	NA	3	AoT
22728111	*Hierococcyx sparverioides*	BodyLength	2	1	ASIZ
22728111	*Hierococcyx sparverioides*	BodyMass	NA	2	HBW
22728111	*Hierococcyx sparverioides*	HeadLength	4	1	ASIZ
22728111	*Hierococcyx sparverioides*	TailLength	5	1	AoT; ASIZ
22728111	*Hierococcyx sparverioides*	TarsusLength	6	1	AoT; ASIZ
22728111	*Hierococcyx sparverioides*	WingLength	7	1	AoT; ASIZ
22728111	*Hierococcyx sparverioides*	ClutchSize	NA	NA	NA
22728111	*Hierococcyx sparverioides*	IncuPeriod	NA	2	HBW
22728111	*Hierococcyx sparverioides*	EggLength	NA	2	HBW
22728111	*Hierococcyx sparverioides*	EggWidth	NA	2	HBW
61450351	*Cuculus saturatus*	BodyLength	3	1	ASIZ
61450351	*Cuculus saturatus*	BodyMass	2	1	ASIZ
61450351	*Cuculus saturatus*	HeadLength	4	1	ASIZ
61450351	*Cuculus saturatus*	TailLength	5	1	AoT; ASIZ
61450351	*Cuculus saturatus*	TarsusLength	6	1	AoT; ASIZ
61450351	*Cuculus saturatus*	WingLength	6	1	AoT; ASIZ
61450351	*Cuculus saturatus*	ClutchSize	NA	3	AoT
61450351	*Cuculus saturatus*	IncuPeriod	NA	NA	NA
61450351	*Cuculus saturatus*	EggLength	NA	3	AoT
61450351	*Cuculus saturatus*	EggWidth	NA	3	AoT
22688522	*Tyto longimembris*	BodyLength	NA	2	HBW
22688522	*Tyto longimembris*	BodyMass	1	1	ASIZ
22688522	*Tyto longimembris*	HeadLength	1	1	ASIZ
22688522	*Tyto longimembris*	TailLength	5	1	AoT; ASIZ
22688522	*Tyto longimembris*	TarsusLength	5	1	AoT; ASIZ
22688522	*Tyto longimembris*	WingLength	4	1	AoT
22688522	*Tyto longimembris*	ClutchSize	3	2	AoT
22688522	*Tyto longimembris*	IncuPeriod	NA	3	HBW
22688522	*Tyto longimembris*	EggLength	NA	3	AoT
22688522	*Tyto longimembris*	EggWidth	NA	3	AoT
22688579	*Otus spilocephalus*	BodyLength	16	1	ASIZ
22688579	*Otus spilocephalus*	BodyMass	18	1	ASIZ; MAPS
22688579	*Otus spilocephalus*	HeadLength	27	1	ASIZ; MAPS
22688579	*Otus spilocephalus*	TailLength	43	1	AoT; ASIZ; MAPS
22688579	*Otus spilocephalus*	TarsusLength	43	1	AoT; ASIZ; MAPS
22688579	*Otus spilocephalus*	WingLength	44	1	AoT; ASIZ; MAPS
22688579	*Otus spilocephalus*	ClutchSize	NA	2	AoT
22688579	*Otus spilocephalus*	IncuPeriod	NA	2	AoT
22688579	*Otus spilocephalus*	EggLength	NA	3	HBW
22688579	*Otus spilocephalus*	EggWidth	NA	3	HBW
61855318	*Otus lettia*	BodyLength	49	1	ASIZ
61855318	*Otus lettia*	BodyMass	31	1	ASIZ; MAPS
61855318	*Otus lettia*	HeadLength	62	1	ASIZ; MAPS
61855318	*Otus lettia*	TailLength	77	1	AoT; ASIZ; MAPS
61855318	*Otus lettia*	TarsusLength	75	1	AoT; ASIZ; MAPS
61855318	*Otus lettia*	WingLength	76	1	AoT; ASIZ; MAPS
61855318	*Otus lettia*	ClutchSize	NA	2	AoT
61855318	*Otus lettia*	IncuPeriod	NA	3	AoT
61855318	*Otus lettia*	EggLength	2	3	AoT
61855318	*Otus lettia*	EggWidth	2	3	AoT
22688651	*Otus elegans*	BodyLength	5	1	ASIZ
22688651	*Otus elegans*	BodyMass	11	1	AoT; ASIZ
22688651	*Otus elegans*	HeadLength	6	1	ASIZ
22688651	*Otus elegans*	TailLength	16	1	AoT; ASIZ
22688651	*Otus elegans*	TarsusLength	16	1	AoT; ASIZ
22688651	*Otus elegans*	WingLength	16	1	AoT; ASIZ
22688651	*Otus elegans*	ClutchSize	NA	2	AoT
22688651	*Otus elegans*	IncuPeriod	NA	3	AoT
22688651	*Otus elegans*	EggLength	NA	3	HBW
22688651	*Otus elegans*	EggWidth	NA	3	HBW
22689017	*Ketupa flavipes*	BodyLength	NA	2	HBW
22689017	*Ketupa flavipes*	BodyMass	NA	3	Elton
22689017	*Ketupa flavipes*	HeadLength	NA	NA	NA
22689017	*Ketupa flavipes*	TailLength	5	1	AoT
22689017	*Ketupa flavipes*	TarsusLength	5	1	AoT
22689017	*Ketupa flavipes*	WingLength	5	1	AoT
22689017	*Ketupa flavipes*	ClutchSize	NA	2	AoT
22689017	*Ketupa flavipes*	IncuPeriod	NA	NA	NA
22689017	*Ketupa flavipes*	EggLength	NA	2	HBW
22689017	*Ketupa flavipes*	EggWidth	NA	2	HBW
22689200	*Glaucidium brodiei*	BodyLength	3	1	ASIZ
22689200	*Glaucidium brodiei*	BodyMass	1	1	ASIZ
22689200	*Glaucidium brodiei*	HeadLength	3	1	ASIZ
22689200	*Glaucidium brodiei*	TailLength	15	1	AoT; ASIZ
22689200	*Glaucidium brodiei*	TarsusLength	15	1	AoT; ASIZ
22689200	*Glaucidium brodiei*	WingLength	15	1	AoT; ASIZ
22689200	*Glaucidium brodiei*	ClutchSize	NA	2	AoT
22689200	*Glaucidium brodiei*	IncuPeriod	NA	3	HBW
22689200	*Glaucidium brodiei*	EggLength	NA	3	HBW
22689200	*Glaucidium brodiei*	EggWidth	NA	3	HBW
22689071	*Strix leptogrammica*	BodyLength	1	1	ASIZ
22689071	*Strix leptogrammica*	BodyMass	1	1	ASIZ
22689071	*Strix leptogrammica*	HeadLength	1	1	ASIZ
22689071	*Strix leptogrammica*	TailLength	5	1	AoT; ASIZ
22689071	*Strix leptogrammica*	TarsusLength	5	1	AoT; ASIZ
22689071	*Strix leptogrammica*	WingLength	5	1	AoT; ASIZ
22689071	*Strix leptogrammica*	ClutchSize	NA	2	HBW
22689071	*Strix leptogrammica*	IncuPeriod	NA	3	HBW
22689071	*Strix leptogrammica*	EggLength	NA	3	HBW
22689071	*Strix leptogrammica*	EggWidth	NA	3	HBW
22725477	*Strix nivicolum*	BodyLength	NA	2	HBW
22725477	*Strix nivicolum*	BodyMass	NA	2	HBW
22725477	*Strix nivicolum*	HeadLength	1	1	ASIZ
22725477	*Strix nivicolum*	TailLength	6	1	AoT; ASIZ
22725477	*Strix nivicolum*	TarsusLength	6	1	AoT; ASIZ
22725477	*Strix nivicolum*	WingLength	6	1	AoT; ASIZ
22725477	*Strix nivicolum*	ClutchSize	NA	2	AoT
22725477	*Strix nivicolum*	IncuPeriod	NA	NA	NA
22725477	*Strix nivicolum*	EggLength	NA	NA	NA
22725477	*Strix nivicolum*	EggWidth	NA	NA	NA
22725653	*Ninox japonica*	BodyLength	13	1	ASIZ
22725653	*Ninox japonica*	BodyMass	10	1	ASIZ
22725653	*Ninox japonica*	HeadLength	17	1	ASIZ
22725653	*Ninox japonica*	TailLength	27	1	AoT; ASIZ
22725653	*Ninox japonica*	TarsusLength	26	1	AoT; ASIZ
22725653	*Ninox japonica*	WingLength	27	1	AoT; ASIZ
22725653	*Ninox japonica*	ClutchSize	NA	2	AoT
22725653	*Ninox japonica*	IncuPeriod	NA	3	HBW
22725653	*Ninox japonica*	EggLength	NA	3	AoT
22725653	*Ninox japonica*	EggWidth	NA	3	AoT
22689985	*Caprimulgus affinis*	BodyLength	10	1	ASIZ
22689985	*Caprimulgus affinis*	BodyMass	3	1	ASIZ
22689985	*Caprimulgus affinis*	HeadLength	13	1	ASIZ
22689985	*Caprimulgus affinis*	TailLength	20	1	AoT; ASIZ
22689985	*Caprimulgus affinis*	TarsusLength	23	1	AoT; ASIZ
22689985	*Caprimulgus affinis*	WingLength	24	1	AoT; ASIZ
22689985	*Caprimulgus affinis*	ClutchSize	NA	3	AoT
22689985	*Caprimulgus affinis*	IncuPeriod	NA	NA	NA
22689985	*Caprimulgus affinis*	EggLength	NA	3	AoT
22689985	*Caprimulgus affinis*	EggWidth	NA	3	AoT
22686684	*Hirundapus cochinchinensis*	BodyLength	1	1	ASIZ
22686684	*Hirundapus cochinchinensis*	BodyMass	1	1	ASIZ
22686684	*Hirundapus cochinchinensis*	HeadLength	1	1	ASIZ
22686684	*Hirundapus cochinchinensis*	TailLength	3	1	AoT; ASIZ
22686684	*Hirundapus cochinchinensis*	TarsusLength	3	1	AoT; ASIZ
22686684	*Hirundapus cochinchinensis*	WingLength	3	1	AoT; ASIZ
22686684	*Hirundapus cochinchinensis*	ClutchSize	NA	NA	NA
22686684	*Hirundapus cochinchinensis*	IncuPeriod	NA	NA	NA
22686684	*Hirundapus cochinchinensis*	EggLength	NA	NA	NA
22686684	*Hirundapus cochinchinensis*	EggWidth	NA	NA	NA
22686861	*Apus nipalensis*	BodyLength	17	1	ASIZ
22686861	*Apus nipalensis*	BodyMass	13	1	AoT; ASIZ
22686861	*Apus nipalensis*	HeadLength	21	1	ASIZ
22686861	*Apus nipalensis*	TailLength	30	1	AoT; ASIZ
22686861	*Apus nipalensis*	TarsusLength	29	1	AoT; ASIZ
22686861	*Apus nipalensis*	WingLength	29	1	AoT; ASIZ
22686861	*Apus nipalensis*	ClutchSize	NA	2	AoT
22686861	*Apus nipalensis*	IncuPeriod	NA	2	AoT
22686861	*Apus nipalensis*	EggLength	NA	3	AoT
22686861	*Apus nipalensis*	EggWidth	NA	3	AoT
22683027	*Alcedo atthis*	BodyLength	10	1	ASIZ
22683027	*Alcedo atthis*	BodyMass	13	1	ASIZ; MAPS
22683027	*Alcedo atthis*	HeadLength	19	1	ASIZ; MAPS
22683027	*Alcedo atthis*	TailLength	38	1	AoT; ASIZ; MAPS
22683027	*Alcedo atthis*	TarsusLength	41	1	AoT; ASIZ; MAPS
22683027	*Alcedo atthis*	WingLength	41	1	AoT; ASIZ; MAPS
22683027	*Alcedo atthis*	ClutchSize	NA	2	AoT
22683027	*Alcedo atthis*	IncuPeriod	NA	2	AoT
22683027	*Alcedo atthis*	EggLength	NA	3	AoT
22683027	*Alcedo atthis*	EggWidth	NA	3	AoT
22734433	*Psilopogon nuchalis*	BodyLength	50	1	ASIZ
22734433	*Psilopogon nuchalis*	BodyMass	49	1	AoT; ASIZ; MAPS
22734433	*Psilopogon nuchalis*	HeadLength	75	1	ASIZ; MAPS
22734433	*Psilopogon nuchalis*	TailLength	79	1	AoT; ASIZ; MAPS
22734433	*Psilopogon nuchalis*	TarsusLength	79	1	AoT; ASIZ; MAPS
22734433	*Psilopogon nuchalis*	WingLength	80	1	AoT; ASIZ; MAPS
22734433	*Psilopogon nuchalis*	ClutchSize	NA	2	AoT
22734433	*Psilopogon nuchalis*	IncuPeriod	NA	2	AoT
22734433	*Psilopogon nuchalis*	EggLength	NA	2	HBW
22734433	*Psilopogon nuchalis*	EggWidth	NA	2	HBW
22681068	*Picoides canicapillus*	BodyLength	NA	2	HBW
22681068	*Picoides canicapillus*	BodyMass	3	1	MAPS
22681068	*Picoides canicapillus*	HeadLength	3	1	MAPS
22681068	*Picoides canicapillus*	TailLength	6	1	AoT; MAPS
22681068	*Picoides canicapillus*	TarsusLength	6	1	AoT; MAPS
22681068	*Picoides canicapillus*	WingLength	6	1	AoT; MAPS
22681068	*Picoides canicapillus*	ClutchSize	NA	2	HBW
22681068	*Picoides canicapillus*	IncuPeriod	NA	2	HBW
22681068	*Picoides canicapillus*	EggLength	NA	NA	NA
22681068	*Picoides canicapillus*	EggWidth	NA	NA	NA
22727124	*Dendrocopos leucotos*	BodyLength	8	1	YIO
22727124	*Dendrocopos leucotos*	BodyMass	NA	2	HBW
22727124	*Dendrocopos leucotos*	HeadLength	8	1	YIO
22727124	*Dendrocopos leucotos*	TailLength	8	1	YIO
22727124	*Dendrocopos leucotos*	TarsusLength	8	1	YIO
22727124	*Dendrocopos leucotos*	WingLength	8	1	YIO
22727124	*Dendrocopos leucotos*	ClutchSize	NA	2	AoT
22727124	*Dendrocopos leucotos*	IncuPeriod	NA	2	AoT
22727124	*Dendrocopos leucotos*	EggLength	NA	NA	NA
22727124	*Dendrocopos leucotos*	EggWidth	NA	NA	NA
22726503	*Picus canus*	BodyLength	8	1	YIO
22726503	*Picus canus*	BodyMass	2	1	AoT; MAPS
22726503	*Picus canus*	HeadLength	9	1	MAPS; YIO
22726503	*Picus canus*	TailLength	11	1	AoT; YIO
22726503	*Picus canus*	TarsusLength	12	1	AoT; MAPS; YIO
22726503	*Picus canus*	WingLength	12	1	AoT; MAPS; YIO
22726503	*Picus canus*	ClutchSize	NA	3	AoT
22726503	*Picus canus*	IncuPeriod	NA	2	AoT
22726503	*Picus canus*	EggLength	NA	3	AoT
22726503	*Picus canus*	EggWidth	NA	3	AoT
45354964	*Falco peregrinus*	BodyLength	2	1	ASIZ
45354964	*Falco peregrinus*	BodyMass	NA	2	HBW
45354964	*Falco peregrinus*	HeadLength	2	1	ASIZ
45354964	*Falco peregrinus*	TailLength	10	1	AoT; ASIZ; TM
45354964	*Falco peregrinus*	TarsusLength	7	1	AoT; ASIZ
45354964	*Falco peregrinus*	WingLength	10	1	AoT; ASIZ; TM
45354964	*Falco peregrinus*	ClutchSize	NA	2	AoT
45354964	*Falco peregrinus*	IncuPeriod	NA	2	HBW
45354964	*Falco peregrinus*	EggLength	NA	NA	NA
45354964	*Falco peregrinus*	EggWidth	NA	NA	NA
22698684	*Pitta nympha*	BodyLength	1	1	ASIZ
22698684	*Pitta nympha*	BodyMass	14	1	AoT; MAPS
22698684	*Pitta nympha*	HeadLength	14	1	ASIZ; MAPS
22698684	*Pitta nympha*	TailLength	27	1	AoT; ASIZ; MAPS
22698684	*Pitta nympha*	TarsusLength	26	1	AoT; ASIZ; MAPS
22698684	*Pitta nympha*	WingLength	27	1	AoT; ASIZ; MAPS
22698684	*Pitta nympha*	ClutchSize	NA	3	AoT
22698684	*Pitta nympha*	IncuPeriod	NA	3	AoT
22698684	*Pitta nympha*	EggLength	NA	NA	NA
22698684	*Pitta nympha*	EggWidth	NA	NA	NA
103694119	*Pericrocotus solaris*	BodyLength	1	1	ASIZ
103694119	*Pericrocotus solaris*	BodyMass	5	1	ASIZ; MAPS
103694119	*Pericrocotus solaris*	HeadLength	4	1	MAPS
103694119	*Pericrocotus solaris*	TailLength	13	1	AoT; ASIZ; MAPS
103694119	*Pericrocotus solaris*	TarsusLength	13	1	AoT; ASIZ; MAPS
103694119	*Pericrocotus solaris*	WingLength	13	1	AoT; ASIZ; MAPS
103694119	*Pericrocotus solaris*	ClutchSize	NA	3	HBW
103694119	*Pericrocotus solaris*	IncuPeriod	NA	NA	NA
103694119	*Pericrocotus solaris*	EggLength	NA	3	HBW
103694119	*Pericrocotus solaris*	EggWidth	NA	3	HBW
22706473	*Coracina macei*	BodyLength	NA	2	HBW
22706473	*Coracina macei*	BodyMass	NA	3	Elton
22706473	*Coracina macei*	HeadLength	NA	NA	NA
22706473	*Coracina macei*	TailLength	2	1	AoT
22706473	*Coracina macei*	TarsusLength	2	1	AoT
22706473	*Coracina macei*	WingLength	2	1	AoT
22706473	*Coracina macei*	ClutchSize	NA	2	HBW
22706473	*Coracina macei*	IncuPeriod	NA	NA	NA
22706473	*Coracina macei*	EggLength	NA	NA	NA
22706473	*Coracina macei*	EggWidth	NA	NA	NA
22705029	*Lanius schach*	BodyLength	4	1	ASIZ
22705029	*Lanius schach*	BodyMass	4	1	ASIZ
22705029	*Lanius schach*	HeadLength	5	1	ASIZ
22705029	*Lanius schach*	TailLength	7	1	AoT; ASIZ
22705029	*Lanius schach*	TarsusLength	9	1	AoT; ASIZ
22705029	*Lanius schach*	WingLength	8	1	AoT; ASIZ
22705029	*Lanius schach*	ClutchSize	NA	2	AoT
22705029	*Lanius schach*	IncuPeriod	NA	2	AoT
22705029	*Lanius schach*	EggLength	NA	NA	NA
22705029	*Lanius schach*	EggWidth	NA	NA	NA
22716753	*Erpornis zantholeuca*	BodyLength	4	1	ASIZ
22716753	*Erpornis zantholeuca*	BodyMass	76	1	AoT; ASIZ; MAPS
22716753	*Erpornis zantholeuca*	HeadLength	58	1	ASIZ; MAPS
22716753	*Erpornis zantholeuca*	TailLength	72	1	AoT; ASIZ; MAPS
22716753	*Erpornis zantholeuca*	TarsusLength	76	1	AoT; ASIZ; MAPS
22716753	*Erpornis zantholeuca*	WingLength	78	1	AoT; ASIZ; MAPS
22716753	*Erpornis zantholeuca*	ClutchSize	NA	2	HBW
22716753	*Erpornis zantholeuca*	IncuPeriod	NA	NA	NA
22716753	*Erpornis zantholeuca*	EggLength	NA	NA	NA
22716753	*Erpornis zantholeuca*	EggWidth	NA	NA	NA
22706394	*Oriolus chinensis*	BodyLength	NA	2	HBW
22706394	*Oriolus chinensis*	BodyMass	NA	2	HBW
22706394	*Oriolus chinensis*	HeadLength	NA	NA	NA
22706394	*Oriolus chinensis*	TailLength	3	1	AoT
22706394	*Oriolus chinensis*	TarsusLength	4	1	AoT
22706394	*Oriolus chinensis*	WingLength	4	1	AoT
22706394	*Oriolus chinensis*	ClutchSize	NA	2	AoT
22706394	*Oriolus chinensis*	IncuPeriod	NA	2	AoT
22706394	*Oriolus chinensis*	EggLength	NA	2	HBW
22706394	*Oriolus chinensis*	EggWidth	NA	2	HBW
22706446	*Oriolus traillii*	BodyLength	8	1	YIO
22706446	*Oriolus traillii*	BodyMass	2	1	MAPS
22706446	*Oriolus traillii*	HeadLength	10	1	MAPS; YIO
22706446	*Oriolus traillii*	TailLength	11	1	AoT; MAPS; YIO
22706446	*Oriolus traillii*	TarsusLength	11	1	AoT; MAPS; YIO
22706446	*Oriolus traillii*	WingLength	11	1	AoT; MAPS; YIO
22706446	*Oriolus traillii*	ClutchSize	NA	2	HBW
22706446	*Oriolus traillii*	IncuPeriod	NA	NA	NA
22706446	*Oriolus traillii*	EggLength	NA	2	HBW
22706446	*Oriolus traillii*	EggWidth	NA	2	HBW
22706961	*Dicrurus macrocercus*	BodyLength	13	1	ASIZ
22706961	*Dicrurus macrocercus*	BodyMass	10	1	ASIZ
22706961	*Dicrurus macrocercus*	HeadLength	17	1	ASIZ
22706961	*Dicrurus macrocercus*	TailLength	25	1	AoT; ASIZ
22706961	*Dicrurus macrocercus*	TarsusLength	27	1	AoT; ASIZ
22706961	*Dicrurus macrocercus*	WingLength	26	1	AoT; ASIZ
22706961	*Dicrurus macrocercus*	ClutchSize	NA	3	AoT
22706961	*Dicrurus macrocercus*	IncuPeriod	NA	2	HBW
22706961	*Dicrurus macrocercus*	EggLength	NA	3	AoT
22706961	*Dicrurus macrocercus*	EggWidth	NA	3	AoT
22706973	*Dicrurus aeneus*	BodyLength	2	1	ASIZ
22706973	*Dicrurus aeneus*	BodyMass	30	1	MAPS
22706973	*Dicrurus aeneus*	HeadLength	27	1	ASIZ; MAPS
22706973	*Dicrurus aeneus*	TailLength	35	1	AoT; ASIZ; MAPS
22706973	*Dicrurus aeneus*	TarsusLength	37	1	AoT; ASIZ; MAPS
22706973	*Dicrurus aeneus*	WingLength	37	1	AoT; ASIZ; MAPS
22706973	*Dicrurus aeneus*	ClutchSize	NA	2	AoT
22706973	*Dicrurus aeneus*	IncuPeriod	NA	3	HBW
22706973	*Dicrurus aeneus*	EggLength	4	3	AoT
22706973	*Dicrurus aeneus*	EggWidth	4	3	AoT
103715755	*Hypothymis azurea*	BodyLength	1	1	ASIZ
103715755	*Hypothymis azurea*	BodyMass	238	1	MAPS
103715755	*Hypothymis azurea*	HeadLength	225	1	MAPS
103715755	*Hypothymis azurea*	TailLength	234	1	AoT; ASIZ; MAPS
103715755	*Hypothymis azurea*	TarsusLength	238	1	AoT; ASIZ; MAPS
103715755	*Hypothymis azurea*	WingLength	248	1	AoT; ASIZ; MAPS
103715755	*Hypothymis azurea*	ClutchSize	NA	3	AoT
103715755	*Hypothymis azurea*	IncuPeriod	NA	3	AoT
103715755	*Hypothymis azurea*	EggLength	NA	2	HBW
103715755	*Hypothymis azurea*	EggWidth	NA	2	HBW
22707151	*Terpsiphone atrocaudata*	BodyLength	2	1	ASIZ
22707151	*Terpsiphone atrocaudata*	BodyMass	14	1	AoT; ASIZ; MAPS
22707151	*Terpsiphone atrocaudata*	HeadLength	8	1	ASIZ; MAPS
22707151	*Terpsiphone atrocaudata*	TailLength	23	1	AoT; ASIZ; MAPS
22707151	*Terpsiphone atrocaudata*	TarsusLength	25	1	AoT; ASIZ; MAPS
22707151	*Terpsiphone atrocaudata*	WingLength	25	1	AoT; ASIZ; MAPS
22707151	*Terpsiphone atrocaudata*	ClutchSize	NA	3	AoT
22707151	*Terpsiphone atrocaudata*	IncuPeriod	NA	NA	NA
22707151	*Terpsiphone atrocaudata*	EggLength	NA	NA	NA
22707151	*Terpsiphone atrocaudata*	EggWidth	NA	NA	NA
103723684	*Garrulus glandarius*	BodyLength	NA	2	HBW
103723684	*Garrulus glandarius*	BodyMass	1	1	MAPS
103723684	*Garrulus glandarius*	HeadLength	1	1	MAPS
103723684	*Garrulus glandarius*	TailLength	7	1	AoT
103723684	*Garrulus glandarius*	TarsusLength	9	1	AoT; MAPS
103723684	*Garrulus glandarius*	WingLength	9	1	AoT; MAPS
103723684	*Garrulus glandarius*	ClutchSize	NA	2	AoT
103723684	*Garrulus glandarius*	IncuPeriod	NA	2	AoT
103723684	*Garrulus glandarius*	EggLength	NA	3	HBW
103723684	*Garrulus glandarius*	EggWidth	NA	3	HBW
22705793	*Urocissa caerulea*	BodyLength	13	1	ASIZ
22705793	*Urocissa caerulea*	BodyMass	7	1	AoT; MAPS
22705793	*Urocissa caerulea*	HeadLength	19	1	ASIZ; MAPS
22705793	*Urocissa caerulea*	TailLength	21	1	AoT; ASIZ
22705793	*Urocissa caerulea*	TarsusLength	24	1	AoT; ASIZ
22705793	*Urocissa caerulea*	WingLength	28	1	AoT; ASIZ
22705793	*Urocissa caerulea*	ClutchSize	NA	2	AoT
22705793	*Urocissa caerulea*	IncuPeriod	NA	3	AoT
22705793	*Urocissa caerulea*	EggLength	NA	NA	NA
22705793	*Urocissa caerulea*	EggWidth	NA	NA	NA
22705839	*Dendrocitta formosae*	BodyLength	4	1	ASIZ
22705839	*Dendrocitta formosae*	BodyMass	10	1	AoT; ASIZ; MAPS
22705839	*Dendrocitta formosae*	HeadLength	11	1	ASIZ; MAPS
22705839	*Dendrocitta formosae*	TailLength	26	1	AoT; ASIZ; MAPS
22705839	*Dendrocitta formosae*	TarsusLength	28	1	AoT; ASIZ; MAPS
22705839	*Dendrocitta formosae*	WingLength	28	1	AoT; ASIZ; MAPS
22705839	*Dendrocitta formosae*	ClutchSize	NA	2	AoT
22705839	*Dendrocitta formosae*	IncuPeriod	NA	NA	NA
22705839	*Dendrocitta formosae*	EggLength	NA	NA	NA
22705839	*Dendrocitta formosae*	EggWidth	NA	NA	NA
103727252	*Nucifraga caryocatactes*	BodyLength	9	1	YIO
103727252	*Nucifraga caryocatactes*	BodyMass	NA	2	HBW
103727252	*Nucifraga caryocatactes*	HeadLength	9	1	YIO
103727252	*Nucifraga caryocatactes*	TailLength	12	1	AoT; YIO
103727252	*Nucifraga caryocatactes*	TarsusLength	12	1	AoT; YIO
103727252	*Nucifraga caryocatactes*	WingLength	12	1	AoT; YIO
103727252	*Nucifraga caryocatactes*	ClutchSize	NA	2	AoT
103727252	*Nucifraga caryocatactes*	IncuPeriod	NA	2	HBW
103727252	*Nucifraga caryocatactes*	EggLength	NA	3	HBW
103727252	*Nucifraga caryocatactes*	EggWidth	NA	3	HBW
103727590	*Corvus macrorhynchos*	BodyLength	1	1	ASIZ
103727590	*Corvus macrorhynchos*	BodyMass	1	1	AoT
103727590	*Corvus macrorhynchos*	HeadLength	1	1	ASIZ
103727590	*Corvus macrorhynchos*	TailLength	9	1	AoT
103727590	*Corvus macrorhynchos*	TarsusLength	9	1	AoT
103727590	*Corvus macrorhynchos*	WingLength	9	1	AoT
103727590	*Corvus macrorhynchos*	ClutchSize	NA	2	AoT
103727590	*Corvus macrorhynchos*	IncuPeriod	NA	2	HBW
103727590	*Corvus macrorhynchos*	EggLength	NA	NA	NA
103727590	*Corvus macrorhynchos*	EggWidth	NA	NA	NA
22717424	*Alauda gulgula*	BodyLength	14	1	ASIZ
22717424	*Alauda gulgula*	BodyMass	6	1	AoT; ASIZ
22717424	*Alauda gulgula*	HeadLength	13	1	ASIZ
22717424	*Alauda gulgula*	TailLength	24	1	AoT; ASIZ
22717424	*Alauda gulgula*	TarsusLength	24	1	AoT; ASIZ
22717424	*Alauda gulgula*	WingLength	24	1	AoT; ASIZ
22717424	*Alauda gulgula*	ClutchSize	NA	2	HBW
22717424	*Alauda gulgula*	IncuPeriod	NA	2	HBW
22717424	*Alauda gulgula*	EggLength	NA	NA	NA
22717424	*Alauda gulgula*	EggWidth	NA	NA	NA
103815539	*Riparia chinensis*	BodyLength	2	1	ASIZ
103815539	*Riparia chinensis*	BodyMass	1	1	AoT
103815539	*Riparia chinensis*	HeadLength	2	1	ASIZ
103815539	*Riparia chinensis*	TailLength	10	1	AoT; ASIZ
103815539	*Riparia chinensis*	TarsusLength	12	1	AoT; ASIZ
103815539	*Riparia chinensis*	WingLength	12	1	AoT; ASIZ
103815539	*Riparia chinensis*	ClutchSize	NA	2	AoT
103815539	*Riparia chinensis*	IncuPeriod	NA	3	AoT
103815539	*Riparia chinensis*	EggLength	NA	3	AoT
103815539	*Riparia chinensis*	EggWidth	NA	3	AoT
22712252	*Hirundo rustica*	BodyLength	14	1	ASIZ
22712252	*Hirundo rustica*	BodyMass	23	1	AoT; ASIZ
22712252	*Hirundo rustica*	HeadLength	15	1	ASIZ
22712252	*Hirundo rustica*	TailLength	33	1	AoT; ASIZ
22712252	*Hirundo rustica*	TarsusLength	36	1	AoT; ASIZ
22712252	*Hirundo rustica*	WingLength	36	1	AoT; ASIZ
22712252	*Hirundo rustica*	ClutchSize	NA	2	AoT
22712252	*Hirundo rustica*	IncuPeriod	NA	2	AoT
22712252	*Hirundo rustica*	EggLength	NA	3	AoT
22712252	*Hirundo rustica*	EggWidth	NA	3	AoT
104006414	*Hirundo tahitica*	BodyLength	2	1	ASIZ
104006414	*Hirundo tahitica*	BodyMass	11	1	AoT; MAPS
104006414	*Hirundo tahitica*	HeadLength	5	1	ASIZ; MAPS
104006414	*Hirundo tahitica*	TailLength	12	1	AoT; ASIZ; MAPS
104006414	*Hirundo tahitica*	TarsusLength	13	1	AoT; ASIZ; MAPS
104006414	*Hirundo tahitica*	WingLength	15	1	AoT; ASIZ; MAPS
104006414	*Hirundo tahitica*	ClutchSize	NA	2	AoT
104006414	*Hirundo tahitica*	IncuPeriod	NA	2	AoT
104006414	*Hirundo tahitica*	EggLength	NA	3	AoT
104006414	*Hirundo tahitica*	EggWidth	NA	3	AoT
103812643	*Cecropis daurica*	BodyLength	1	1	ASIZ
103812643	*Cecropis daurica*	BodyMass	6	1	AoT; ASIZ
103812643	*Cecropis daurica*	HeadLength	1	1	ASIZ
103812643	*Cecropis daurica*	TailLength	7	1	AoT
103812643	*Cecropis daurica*	TarsusLength	8	1	AoT; ASIZ
103812643	*Cecropis daurica*	WingLength	8	1	AoT; ASIZ
103812643	*Cecropis daurica*	ClutchSize	NA	2	AoT
103812643	*Cecropis daurica*	IncuPeriod	NA	2	HBW
103812643	*Cecropis daurica*	EggLength	NA	3	AoT
103812643	*Cecropis daurica*	EggWidth	NA	3	AoT
22712491	*Delichon dasypus*	BodyLength	NA	2	HBW
22712491	*Delichon dasypus*	BodyMass	NA	3	HBW
22712491	*Delichon dasypus*	HeadLength	1	1	ASIZ
22712491	*Delichon dasypus*	TailLength	3	1	AoT; ASIZ
22712491	*Delichon dasypus*	TarsusLength	2	1	AoT
22712491	*Delichon dasypus*	WingLength	3	1	AoT; ASIZ
22712491	*Delichon dasypus*	ClutchSize	NA	2	AoT
22712491	*Delichon dasypus*	IncuPeriod	NA	NA	NA
22712491	*Delichon dasypus*	EggLength	NA	3	AoT
22712491	*Delichon dasypus*	EggWidth	NA	3	AoT
22735965	*Periparus ater*	BodyLength	NA	2	HBW
22735965	*Periparus ater*	BodyMass	11	1	MAPS
22735965	*Periparus ater*	HeadLength	6	1	MAPS
22735965	*Periparus ater*	TailLength	21	1	AoT; MAPS
22735965	*Periparus ater*	TarsusLength	16	1	AoT; MAPS
22735965	*Periparus ater*	WingLength	21	1	AoT; MAPS
22735965	*Periparus ater*	ClutchSize	NA	3	AoT
22735965	*Periparus ater*	IncuPeriod	NA	2	HBW
22735965	*Periparus ater*	EggLength	NA	NA	NA
22735965	*Periparus ater*	EggWidth	NA	NA	NA
103758936	*Sittiparus castaneoventris*	BodyLength	1	1	ASIZ
103758936	*Sittiparus castaneoventris*	BodyMass	1	1	AoT
103758936	*Sittiparus castaneoventris*	HeadLength	NA	NA	NA
103758936	*Sittiparus castaneoventris*	TailLength	10	1	AoT
103758936	*Sittiparus castaneoventris*	TarsusLength	9	1	AoT
103758936	*Sittiparus castaneoventris*	WingLength	10	1	AoT
103758936	*Sittiparus castaneoventris*	ClutchSize	NA	3	AoT
103758936	*Sittiparus castaneoventris*	IncuPeriod	NA	3	AoT
103758936	*Sittiparus castaneoventris*	EggLength	NA	NA	NA
103758936	*Sittiparus castaneoventris*	EggWidth	NA	NA	NA
22711919	*Parus monticolus*	BodyLength	6	1	ASIZ
22711919	*Parus monticolus*	BodyMass	18	1	AoT; MAPS
22711919	*Parus monticolus*	HeadLength	18	1	ASIZ; MAPS
22711919	*Parus monticolus*	TailLength	30	1	AoT; MAPS
22711919	*Parus monticolus*	TarsusLength	29	1	AoT; MAPS
22711919	*Parus monticolus*	WingLength	31	1	AoT; MAPS
22711919	*Parus monticolus*	ClutchSize	NA	3	AoT
22711919	*Parus monticolus*	IncuPeriod	NA	2	AoT
22711919	*Parus monticolus*	EggLength	NA	NA	NA
22711919	*Parus monticolus*	EggWidth	NA	NA	NA
22711939	*Machlolophus holsti*	BodyLength	4	1	ASIZ
22711939	*Machlolophus holsti*	BodyMass	9	1	AoT; ASIZ; MAPS
22711939	*Machlolophus holsti*	HeadLength	6	1	ASIZ; MAPS
22711939	*Machlolophus holsti*	TailLength	17	1	AoT; MAPS
22711939	*Machlolophus holsti*	TarsusLength	17	1	AoT; MAPS
22711939	*Machlolophus holsti*	WingLength	18	1	AoT; MAPS
22711939	*Machlolophus holsti*	ClutchSize	NA	3	AoT
22711939	*Machlolophus holsti*	IncuPeriod	NA	NA	NA
22711939	*Machlolophus holsti*	EggLength	NA	NA	NA
22711939	*Machlolophus holsti*	EggWidth	NA	NA	NA
103870880	*Aegithalos concinnus*	BodyLength	4	1	ASIZ
103870880	*Aegithalos concinnus*	BodyMass	19	1	MAPS
103870880	*Aegithalos concinnus*	HeadLength	13	1	ASIZ; MAPS
103870880	*Aegithalos concinnus*	TailLength	30	1	AoT; ASIZ; MAPS
103870880	*Aegithalos concinnus*	TarsusLength	21	1	AoT; ASIZ; MAPS
103870880	*Aegithalos concinnus*	WingLength	30	1	AoT; ASIZ; MAPS
103870880	*Aegithalos concinnus*	ClutchSize	NA	3	AoT
103870880	*Aegithalos concinnus*	IncuPeriod	NA	3	AoT
103870880	*Aegithalos concinnus*	EggLength	NA	3	HBW
103870880	*Aegithalos concinnus*	EggWidth	NA	3	HBW
103879804	*Sitta europaea*	BodyLength	1	1	ASIZ
103879804	*Sitta europaea*	BodyMass	2	1	MAPS
103879804	*Sitta europaea*	HeadLength	3	1	ASIZ; MAPS
103879804	*Sitta europaea*	TailLength	10	1	AoT; ASIZ; MAPS
103879804	*Sitta europaea*	TarsusLength	10	1	AoT; ASIZ; MAPS
103879804	*Sitta europaea*	WingLength	10	1	AoT; ASIZ; MAPS
103879804	*Sitta europaea*	ClutchSize	NA	2	AoT
103879804	*Sitta europaea*	IncuPeriod	NA	3	AoT
103879804	*Sitta europaea*	EggLength	NA	3	HBW
103879804	*Sitta europaea*	EggWidth	NA	3	HBW
103883277	*Troglodytes troglodytes*	BodyLength	8	1	YIO
103883277	*Troglodytes troglodytes*	BodyMass	17	1	MAPS
103883277	*Troglodytes troglodytes*	HeadLength	20	1	MAPS; YIO
103883277	*Troglodytes troglodytes*	TailLength	25	1	MAPS; YIO
103883277	*Troglodytes troglodytes*	TarsusLength	20	1	MAPS; YIO
103883277	*Troglodytes troglodytes*	WingLength	26	1	MAPS; YIO
103883277	*Troglodytes troglodytes*	ClutchSize	NA	2	AoT
103883277	*Troglodytes troglodytes*	IncuPeriod	NA	3	HBW
103883277	*Troglodytes troglodytes*	EggLength	3	3	AoT
103883277	*Troglodytes troglodytes*	EggWidth	3	3	AoT
22708160	*Cinclus pallasii*	BodyLength	2	1	ASIZ
22708160	*Cinclus pallasii*	BodyMass	4	1	HBW
22708160	*Cinclus pallasii*	HeadLength	2	1	ASIZ
22708160	*Cinclus pallasii*	TailLength	12	1	AoT; ASIZ
22708160	*Cinclus pallasii*	TarsusLength	12	1	AoT; ASIZ
22708160	*Cinclus pallasii*	WingLength	12	1	AoT; ASIZ
22708160	*Cinclus pallasii*	ClutchSize	NA	2	AoT
22708160	*Cinclus pallasii*	IncuPeriod	NA	2	AoT
22708160	*Cinclus pallasii*	EggLength	3	1	AoT
22708160	*Cinclus pallasii*	EggWidth	3	1	AoT
22712600	*Spizixos semitorques*	BodyLength	3	1	ASIZ
22712600	*Spizixos semitorques*	BodyMass	34	1	AoT; MAPS
22712600	*Spizixos semitorques*	HeadLength	33	1	ASIZ; MAPS
22712600	*Spizixos semitorques*	TailLength	45	1	AoT; ASIZ; MAPS
22712600	*Spizixos semitorques*	TarsusLength	45	1	AoT; ASIZ; MAPS
22712600	*Spizixos semitorques*	WingLength	48	1	AoT; ASIZ; MAPS
22712600	*Spizixos semitorques*	ClutchSize	NA	2	AoT
22712600	*Spizixos semitorques*	IncuPeriod	NA	NA	NA
22712600	*Spizixos semitorques*	EggLength	NA	NA	NA
22712600	*Spizixos semitorques*	EggWidth	NA	NA	NA
22712646	*Pycnonotus taivanus*	BodyLength	12	1	ASIZ
22712646	*Pycnonotus taivanus*	BodyMass	9	1	AoT; ASIZ
22712646	*Pycnonotus taivanus*	HeadLength	22	1	ASIZ
22712646	*Pycnonotus taivanus*	TailLength	28	1	AoT; ASIZ
22712646	*Pycnonotus taivanus*	TarsusLength	28	1	AoT; ASIZ
22712646	*Pycnonotus taivanus*	WingLength	27	1	AoT; ASIZ
22712646	*Pycnonotus taivanus*	ClutchSize	NA	2	AoT
22712646	*Pycnonotus taivanus*	IncuPeriod	NA	3	AoT
22712646	*Pycnonotus taivanus*	EggLength	NA	NA	NA
22712646	*Pycnonotus taivanus*	EggWidth	NA	NA	NA
22712643	*Pycnonotus sinensis*	BodyLength	52	1	ASIZ
22712643	*Pycnonotus sinensis*	BodyMass	282	1	AoT; ASIZ; MAPS
22712643	*Pycnonotus sinensis*	HeadLength	261	1	ASIZ; MAPS
22712643	*Pycnonotus sinensis*	TailLength	274	1	AoT; ASIZ; MAPS
22712643	*Pycnonotus sinensis*	TarsusLength	278	1	AoT; ASIZ; MAPS
22712643	*Pycnonotus sinensis*	WingLength	312	1	AoT; ASIZ; MAPS
22712643	*Pycnonotus sinensis*	ClutchSize	NA	2	AoT
22712643	*Pycnonotus sinensis*	IncuPeriod	NA	3	AoT
22712643	*Pycnonotus sinensis*	EggLength	NA	NA	NA
22712643	*Pycnonotus sinensis*	EggWidth	NA	NA	NA
103823996	*Hypsipetes leucocephalus*	BodyLength	14	1	ASIZ
103823996	*Hypsipetes leucocephalus*	BodyMass	58	1	AoT; MAPS
103823996	*Hypsipetes leucocephalus*	HeadLength	61	1	ASIZ; MAPS
103823996	*Hypsipetes leucocephalus*	TailLength	63	1	AoT; ASIZ; MAPS
103823996	*Hypsipetes leucocephalus*	TarsusLength	68	1	AoT; ASIZ; MAPS
103823996	*Hypsipetes leucocephalus*	WingLength	66	1	AoT; ASIZ; MAPS
103823996	*Hypsipetes leucocephalus*	ClutchSize	NA	2	AoT
103823996	*Hypsipetes leucocephalus*	IncuPeriod	NA	NA	NA
103823996	*Hypsipetes leucocephalus*	EggLength	NA	NA	NA
103823996	*Hypsipetes leucocephalus*	EggWidth	NA	NA	NA
22713192	*Hypsipetes amaurotis*	BodyLength	2	1	ASIZ
22713192	*Hypsipetes amaurotis*	BodyMass	12	1	AoT; ASIZ
22713192	*Hypsipetes amaurotis*	HeadLength	3	1	ASIZ
22713192	*Hypsipetes amaurotis*	TailLength	13	1	AoT; ASIZ
22713192	*Hypsipetes amaurotis*	TarsusLength	13	1	AoT; ASIZ
22713192	*Hypsipetes amaurotis*	WingLength	13	1	AoT; ASIZ
22713192	*Hypsipetes amaurotis*	ClutchSize	NA	3	AoT
22713192	*Hypsipetes amaurotis*	IncuPeriod	NA	3	AoT
22713192	*Hypsipetes amaurotis*	EggLength	NA	NA	NA
22713192	*Hypsipetes amaurotis*	EggWidth	NA	NA	NA
22712580	*Regulus goodfellowi*	BodyLength	NA	3	HBW
22712580	*Regulus goodfellowi*	BodyMass	47	1	AoT; MAPS
22712580	*Regulus goodfellowi*	HeadLength	17	1	MAPS
22712580	*Regulus goodfellowi*	TailLength	46	1	AoT; MAPS
22712580	*Regulus goodfellowi*	TarsusLength	26	1	AoT; MAPS
22712580	*Regulus goodfellowi*	WingLength	49	1	AoT; MAPS
22712580	*Regulus goodfellowi*	ClutchSize	NA	2	AoT
22712580	*Regulus goodfellowi*	IncuPeriod	NA	NA	NA
22712580	*Regulus goodfellowi*	EggLength	2	1	AoT
22712580	*Regulus goodfellowi*	EggWidth	2	1	AoT
22734538	*Pnoepyga formosana*	BodyLength	NA	2	HBW
22734538	*Pnoepyga formosana*	BodyMass	19	1	AoT; MAPS
22734538	*Pnoepyga formosana*	HeadLength	11	1	ASIZ; MAPS
22734538	*Pnoepyga formosana*	TailLength	19	1	AoT; ASIZ; MAPS
22734538	*Pnoepyga formosana*	TarsusLength	19	1	AoT; ASIZ; MAPS
22734538	*Pnoepyga formosana*	WingLength	21	1	AoT; ASIZ; MAPS
22734538	*Pnoepyga formosana*	ClutchSize	NA	3	AoT
22734538	*Pnoepyga formosana*	IncuPeriod	NA	NA	NA
22734538	*Pnoepyga formosana*	EggLength	NA	3	AoT
22734538	*Pnoepyga formosana*	EggWidth	NA	3	AoT
22715451	*Abroscopus albogularis*	BodyLength	10	1	ASIZ
22715451	*Abroscopus albogularis*	BodyMass	140	1	AoT; ASIZ; MAPS
22715451	*Abroscopus albogularis*	HeadLength	97	1	ASIZ; MAPS
22715451	*Abroscopus albogularis*	TailLength	131	1	AoT; ASIZ; MAPS
22715451	*Abroscopus albogularis*	TarsusLength	111	1	AoT; ASIZ; MAPS
22715451	*Abroscopus albogularis*	WingLength	142	1	AoT; ASIZ; MAPS
22715451	*Abroscopus albogularis*	ClutchSize	NA	2	AoT
22715451	*Abroscopus albogularis*	IncuPeriod	NA	NA	NA
22715451	*Abroscopus albogularis*	EggLength	NA	3	AoT
22715451	*Abroscopus albogularis*	EggWidth	NA	3	AoT
22714408	*Horornis fortipes*	BodyLength	NA	2	HBW
22714408	*Horornis fortipes*	BodyMass	9	1	AoT
22714408	*Horornis fortipes*	HeadLength	NA	NA	NA
22714408	*Horornis fortipes*	TailLength	9	1	AoT
22714408	*Horornis fortipes*	TarsusLength	9	1	AoT
22714408	*Horornis fortipes*	WingLength	9	1	AoT
22714408	*Horornis fortipes*	ClutchSize	NA	3	AoT
22714408	*Horornis fortipes*	IncuPeriod	NA	NA	NA
22714408	*Horornis fortipes*	EggLength	NA	3	AoT
22714408	*Horornis fortipes*	EggWidth	NA	3	AoT
22735889	*Horornis acanthizoides*	BodyLength	NA	2	HBW
22735889	*Horornis acanthizoides*	BodyMass	118	1	AoT; MAPS
22735889	*Horornis acanthizoides*	HeadLength	66	1	MAPS
22735889	*Horornis acanthizoides*	TailLength	103	1	AoT; MAPS
22735889	*Horornis acanthizoides*	TarsusLength	80	1	AoT; MAPS
22735889	*Horornis acanthizoides*	WingLength	117	1	AoT; MAPS
22735889	*Horornis acanthizoides*	ClutchSize	NA	3	AoT
22735889	*Horornis acanthizoides*	IncuPeriod	NA	NA	NA
22735889	*Horornis acanthizoides*	EggLength	NA	3	AoT
22735889	*Horornis acanthizoides*	EggWidth	NA	3	AoT
22732064	*Locustella alishanensis*	BodyLength	NA	3	HBW
22732064	*Locustella alishanensis*	BodyMass	2	1	AoT
22732064	*Locustella alishanensis*	HeadLength	NA	NA	NA
22732064	*Locustella alishanensis*	TailLength	5	1	AoT
22732064	*Locustella alishanensis*	TarsusLength	5	1	AoT
22732064	*Locustella alishanensis*	WingLength	5	1	AoT
22732064	*Locustella alishanensis*	ClutchSize	NA	3	AoT
22732064	*Locustella alishanensis*	IncuPeriod	NA	NA	NA
22732064	*Locustella alishanensis*	EggLength	NA	3	AoT
22732064	*Locustella alishanensis*	EggWidth	NA	3	AoT
22713491	*Cisticola juncidis*	BodyLength	1	1	ASIZ
22713491	*Cisticola juncidis*	BodyMass	4	1	AoT
22713491	*Cisticola juncidis*	HeadLength	NA	NA	NA
22713491	*Cisticola juncidis*	TailLength	5	1	AoT; ASIZ
22713491	*Cisticola juncidis*	TarsusLength	6	1	AoT; ASIZ
22713491	*Cisticola juncidis*	WingLength	6	1	AoT; ASIZ
22713491	*Cisticola juncidis*	ClutchSize	NA	2	AoT
22713491	*Cisticola juncidis*	IncuPeriod	NA	2	HBW
22713491	*Cisticola juncidis*	EggLength	NA	3	AoT
22713491	*Cisticola juncidis*	EggWidth	NA	3	AoT
22713544	*Cisticola exilis*	BodyLength	3	1	ASIZ
22713544	*Cisticola exilis*	BodyMass	5	1	AoT; ASIZ
22713544	*Cisticola exilis*	HeadLength	1	1	ASIZ
22713544	*Cisticola exilis*	TailLength	12	1	AoT; ASIZ
22713544	*Cisticola exilis*	TarsusLength	12	1	AoT; ASIZ
22713544	*Cisticola exilis*	WingLength	12	1	AoT; ASIZ
22713544	*Cisticola exilis*	ClutchSize	NA	2	AoT
22713544	*Cisticola exilis*	IncuPeriod	NA	2	AoT
22713544	*Cisticola exilis*	EggLength	NA	3	AoT
22713544	*Cisticola exilis*	EggWidth	NA	3	AoT
22713564	*Prinia crinigera*	BodyLength	NA	3	HBW
22713564	*Prinia crinigera*	BodyMass	10	1	AoT; MAPS
22713564	*Prinia crinigera*	HeadLength	3	1	MAPS
22713564	*Prinia crinigera*	TailLength	10	1	AoT; MAPS
22713564	*Prinia crinigera*	TarsusLength	13	1	AoT; MAPS
22713564	*Prinia crinigera*	WingLength	14	1	AoT; MAPS
22713564	*Prinia crinigera*	ClutchSize	NA	2	AoT
22713564	*Prinia crinigera*	IncuPeriod	NA	2	HBW
22713564	*Prinia crinigera*	EggLength	10	3	AoT
22713564	*Prinia crinigera*	EggWidth	10	3	AoT
103777145	*Prinia flaviventris*	BodyLength	4	1	ASIZ
103777145	*Prinia flaviventris*	BodyMass	106	1	AoT; ASIZ; MAPS
103777145	*Prinia flaviventris*	HeadLength	74	1	ASIZ; MAPS
103777145	*Prinia flaviventris*	TailLength	82	1	AoT; ASIZ; MAPS
103777145	*Prinia flaviventris*	TarsusLength	97	1	AoT; ASIZ; MAPS
103777145	*Prinia flaviventris*	WingLength	105	1	AoT; ASIZ; MAPS
103777145	*Prinia flaviventris*	ClutchSize	NA	2	AoT
103777145	*Prinia flaviventris*	IncuPeriod	NA	2	HBW
103777145	*Prinia flaviventris*	EggLength	NA	3	AoT
103777145	*Prinia flaviventris*	EggWidth	NA	3	AoT
22713615	*Prinia inornata*	BodyLength	4	1	ASIZ
22713615	*Prinia inornata*	BodyMass	68	1	AoT; ASIZ; MAPS
22713615	*Prinia inornata*	HeadLength	49	1	ASIZ; MAPS
22713615	*Prinia inornata*	TailLength	60	1	AoT; ASIZ; MAPS
22713615	*Prinia inornata*	TarsusLength	68	1	AoT; ASIZ; MAPS
22713615	*Prinia inornata*	WingLength	68	1	AoT; ASIZ; MAPS
22713615	*Prinia inornata*	ClutchSize	NA	3	AoT
22713615	*Prinia inornata*	IncuPeriod	NA	2	HBW
22713615	*Prinia inornata*	EggLength	NA	3	AoT
22713615	*Prinia inornata*	EggWidth	NA	3	AoT
22734603	*Fulvetta formosana*	BodyLength	NA	3	HBW
22734603	*Fulvetta formosana*	BodyMass	154	1	AoT; MAPS
22734603	*Fulvetta formosana*	HeadLength	84	1	MAPS
22734603	*Fulvetta formosana*	TailLength	133	1	AoT; MAPS
22734603	*Fulvetta formosana*	TarsusLength	100	1	AoT; MAPS
22734603	*Fulvetta formosana*	WingLength	147	1	AoT; MAPS
22734603	*Fulvetta formosana*	ClutchSize	NA	3	AoT
22734603	*Fulvetta formosana*	IncuPeriod	NA	NA	NA
22734603	*Fulvetta formosana*	EggLength	2	1	AoT
22734603	*Fulvetta formosana*	EggWidth	2	1	AoT
22716804	*Sinosuthora webbiana*	BodyLength	28	1	ASIZ
22716804	*Sinosuthora webbiana*	BodyMass	78	1	AoT; ASIZ; MAPS
22716804	*Sinosuthora webbiana*	HeadLength	93	1	ASIZ; MAPS
22716804	*Sinosuthora webbiana*	TailLength	84	1	AoT; ASIZ; MAPS
22716804	*Sinosuthora webbiana*	TarsusLength	99	1	AoT; ASIZ; MAPS
22716804	*Sinosuthora webbiana*	WingLength	97	1	AoT; ASIZ; MAPS
22716804	*Sinosuthora webbiana*	ClutchSize	NA	3	AoT
22716804	*Sinosuthora webbiana*	IncuPeriod	NA	3	AoT
22716804	*Sinosuthora webbiana*	EggLength	NA	NA	NA
22716804	*Sinosuthora webbiana*	EggWidth	NA	NA	NA
22716829	*Suthora verreauxi*	BodyLength	NA	3	HBW
22716829	*Suthora verreauxi*	BodyMass	121	1	MAPS
22716829	*Suthora verreauxi*	HeadLength	44	1	MAPS
22716829	*Suthora verreauxi*	TailLength	108	1	AoT; MAPS
22716829	*Suthora verreauxi*	TarsusLength	57	1	AoT; MAPS
22716829	*Suthora verreauxi*	WingLength	121	1	AoT; MAPS
22716829	*Suthora verreauxi*	ClutchSize	NA	2	AoT
22716829	*Suthora verreauxi*	IncuPeriod	NA	NA	NA
22716829	*Suthora verreauxi*	EggLength	NA	3	AoT
22716829	*Suthora verreauxi*	EggWidth	NA	3	AoT
22716746	*Yuhina brunneiceps*	BodyLength	4	1	ASIZ
22716746	*Yuhina brunneiceps*	BodyMass	182	1	AoT; MAPS
22716746	*Yuhina brunneiceps*	HeadLength	132	1	ASIZ; MAPS
22716746	*Yuhina brunneiceps*	TailLength	185	1	AoT; ASIZ; MAPS
22716746	*Yuhina brunneiceps*	TarsusLength	149	1	AoT; ASIZ; MAPS
22716746	*Yuhina brunneiceps*	WingLength	182	1	AoT; ASIZ; MAPS
22716746	*Yuhina brunneiceps*	ClutchSize	NA	2	AoT
22716746	*Yuhina brunneiceps*	IncuPeriod	NA	3	HBW
22716746	*Yuhina brunneiceps*	EggLength	12	3	AoT
22716746	*Yuhina brunneiceps*	EggWidth	12	3	AoT
22714033	*Zosterops japonicus*	BodyLength	20	1	ASIZ
22714033	*Zosterops japonicus*	BodyMass	128	1	ASIZ; MAPS
22714033	*Zosterops japonicus*	HeadLength	103	1	ASIZ; MAPS
22714033	*Zosterops japonicus*	TailLength	116	1	AoT; ASIZ; MAPS
22714033	*Zosterops japonicus*	TarsusLength	117	1	AoT; ASIZ; MAPS
22714033	*Zosterops japonicus*	WingLength	143	1	AoT; ASIZ; MAPS
22714033	*Zosterops japonicus*	ClutchSize	NA	3	AoT
22714033	*Zosterops japonicus*	IncuPeriod	NA	3	HBW
22714033	*Zosterops japonicus*	EggLength	8	3	AoT
22714033	*Zosterops japonicus*	EggWidth	8	3	AoT
22714037	*Zosterops meyeni*	BodyLength	NA	2	HBW
22714037	*Zosterops meyeni*	BodyMass	NA	3	HBW
22714037	*Zosterops meyeni*	HeadLength	NA	NA	NA
22714037	*Zosterops meyeni*	TailLength	10	1	AoT
22714037	*Zosterops meyeni*	TarsusLength	10	1	AoT
22714037	*Zosterops meyeni*	WingLength	10	1	AoT
22714037	*Zosterops meyeni*	ClutchSize	NA	2	AoT
22714037	*Zosterops meyeni*	IncuPeriod	NA	NA	NA
22714037	*Zosterops meyeni*	EggLength	NA	3	AoT
22714037	*Zosterops meyeni*	EggWidth	NA	3	AoT
22716187	*Cyanoderma ruficeps*	BodyLength	5	1	ASIZ
22716187	*Cyanoderma ruficeps*	BodyMass	1183	1	AoT; ASIZ; MAPS
22716187	*Cyanoderma ruficeps*	HeadLength	1000	1	ASIZ; MAPS
22716187	*Cyanoderma ruficeps*	TailLength	1051	1	AoT; ASIZ; MAPS
22716187	*Cyanoderma ruficeps*	TarsusLength	1039	1	AoT; ASIZ; MAPS
22716187	*Cyanoderma ruficeps*	WingLength	1167	1	AoT; ASIZ; MAPS
22716187	*Cyanoderma ruficeps*	ClutchSize	NA	2	AoT
22716187	*Cyanoderma ruficeps*	IncuPeriod	NA	NA	NA
22716187	*Cyanoderma ruficeps*	EggLength	NA	3	AoT
22716187	*Cyanoderma ruficeps*	EggWidth	NA	3	AoT
22734518	*Pomatorhinus musicus*	BodyLength	6	1	ASIZ
22734518	*Pomatorhinus musicus*	BodyMass	357	1	AoT; ASIZ; MAPS
22734518	*Pomatorhinus musicus*	HeadLength	319	1	ASIZ; MAPS
22734518	*Pomatorhinus musicus*	TailLength	326	1	AoT; ASIZ; MAPS
22734518	*Pomatorhinus musicus*	TarsusLength	343	1	AoT; ASIZ; MAPS
22734518	*Pomatorhinus musicus*	WingLength	358	1	ASIZ; MAPS
22734518	*Pomatorhinus musicus*	ClutchSize	NA	3	AoT
22734518	*Pomatorhinus musicus*	IncuPeriod	NA	NA	NA
22734518	*Pomatorhinus musicus*	EggLength	7	3	AoT
22734518	*Pomatorhinus musicus*	EggWidth	7	3	AoT
22735573	*Erythrogenys erythrocnemis*	BodyLength	4	1	ASIZ
22735573	*Erythrogenys erythrocnemis*	BodyMass	48	1	AoT; ASIZ; MAPS
22735573	*Erythrogenys erythrocnemis*	HeadLength	41	1	ASIZ; MAPS
22735573	*Erythrogenys erythrocnemis*	TailLength	42	1	AoT; ASIZ; MAPS
22735573	*Erythrogenys erythrocnemis*	TarsusLength	49	1	AoT; ASIZ; MAPS
22735573	*Erythrogenys erythrocnemis*	WingLength	46	1	AoT; ASIZ; MAPS
22735573	*Erythrogenys erythrocnemis*	ClutchSize	NA	2	AoT
22735573	*Erythrogenys erythrocnemis*	IncuPeriod	NA	NA	NA
22735573	*Erythrogenys erythrocnemis*	EggLength	NA	3	AoT
22735573	*Erythrogenys erythrocnemis*	EggWidth	NA	3	AoT
22716624	*Schoeniparus brunneus*	BodyLength	3	1	ASIZ
22716624	*Schoeniparus brunneus*	BodyMass	706	1	AoT; ASIZ; MAPS
22716624	*Schoeniparus brunneus*	HeadLength	602	1	ASIZ; MAPS
22716624	*Schoeniparus brunneus*	TailLength	613	1	AoT; ASIZ; MAPS
22716624	*Schoeniparus brunneus*	TarsusLength	628	1	AoT; ASIZ; MAPS
22716624	*Schoeniparus brunneus*	WingLength	673	1	AoT; ASIZ; MAPS
22716624	*Schoeniparus brunneus*	ClutchSize	NA	2	AoT
22716624	*Schoeniparus brunneus*	IncuPeriod	NA	NA	NA
22716624	*Schoeniparus brunneus*	EggLength	8	3	AoT
22716624	*Schoeniparus brunneus*	EggWidth	8	3	AoT
22716644	*Alcippe morrisonia*	BodyLength	10	1	ASIZ
22716644	*Alcippe morrisonia*	BodyMass	1276	1	AoT; ASIZ; MAPS
22716644	*Alcippe morrisonia*	HeadLength	1113	1	ASIZ; MAPS
22716644	*Alcippe morrisonia*	TailLength	1127	1	AoT; ASIZ; MAPS
22716644	*Alcippe morrisonia*	TarsusLength	1175	1	AoT; ASIZ; MAPS
22716644	*Alcippe morrisonia*	WingLength	1263	1	AoT; ASIZ; MAPS
22716644	*Alcippe morrisonia*	ClutchSize	NA	2	AoT
22716644	*Alcippe morrisonia*	IncuPeriod	NA	2	HBW
22716644	*Alcippe morrisonia*	EggLength	3	1	AoT
22716644	*Alcippe morrisonia*	EggWidth	3	1	AoT
22734473	*Garrulax taewanus*	BodyLength	6	1	ASIZ
22734473	*Garrulax taewanus*	BodyMass	7	1	AoT; ASIZ
22734473	*Garrulax taewanus*	HeadLength	9	1	ASIZ
22734473	*Garrulax taewanus*	TailLength	13	1	AoT; ASIZ
22734473	*Garrulax taewanus*	TarsusLength	13	1	AoT; ASIZ; MAPS
22734473	*Garrulax taewanus*	WingLength	14	1	AoT; ASIZ; MAPS
22734473	*Garrulax taewanus*	ClutchSize	NA	2	AoT
22734473	*Garrulax taewanus*	IncuPeriod	NA	3	HBW
22734473	*Garrulax taewanus*	EggLength	21	3	AoT
22734473	*Garrulax taewanus*	EggWidth	21	3	AoT
22734443	*Garrulax ruficeps*	BodyLength	NA	2	HBW
22734443	*Garrulax ruficeps*	BodyMass	2	1	AoT; MAPS
22734443	*Garrulax ruficeps*	HeadLength	1	1	MAPS
22734443	*Garrulax ruficeps*	TailLength	3	1	AoT; MAPS
22734443	*Garrulax ruficeps*	TarsusLength	3	1	AoT; MAPS
22734443	*Garrulax ruficeps*	WingLength	3	1	AoT; MAPS
22734443	*Garrulax ruficeps*	ClutchSize	NA	NA	NA
22734443	*Garrulax ruficeps*	IncuPeriod	NA	NA	NA
22734443	*Garrulax ruficeps*	EggLength	NA	NA	NA
22734443	*Garrulax ruficeps*	EggWidth	NA	NA	NA
22735107	*Garrulax poecilorhynchus*	BodyLength	1	1	ASIZ
22735107	*Garrulax poecilorhynchus*	BodyMass	14	1	AoT; ASIZ; MAPS
22735107	*Garrulax poecilorhynchus*	HeadLength	5	1	ASIZ; MAPS
22735107	*Garrulax poecilorhynchus*	TailLength	13	1	AoT; ASIZ; MAPS
22735107	*Garrulax poecilorhynchus*	TarsusLength	14	1	AoT; ASIZ; MAPS
22735107	*Garrulax poecilorhynchus*	WingLength	14	1	AoT; ASIZ; MAPS
22735107	*Garrulax poecilorhynchus*	ClutchSize	NA	2	AoT
22735107	*Garrulax poecilorhynchus*	IncuPeriod	NA	NA	NA
22735107	*Garrulax poecilorhynchus*	EggLength	3	3	AoT
22735107	*Garrulax poecilorhynchus*	EggWidth	3	3	AoT
22715750	*Trochalopteron morrisonianum*	BodyLength	1	1	ASIZ
22715750	*Trochalopteron morrisonianum*	BodyMass	79	1	AoT; ASIZ; MAPS
22715750	*Trochalopteron morrisonianum*	HeadLength	60	1	ASIZ; MAPS
22715750	*Trochalopteron morrisonianum*	TailLength	67	1	AoT; ASIZ; MAPS
22715750	*Trochalopteron morrisonianum*	TarsusLength	69	1	AoT; ASIZ; MAPS
22715750	*Trochalopteron morrisonianum*	WingLength	75	1	AoT; ASIZ; MAPS
22715750	*Trochalopteron morrisonianum*	ClutchSize	NA	3	AoT
22715750	*Trochalopteron morrisonianum*	IncuPeriod	NA	NA	NA
22715750	*Trochalopteron morrisonianum*	EggLength	NA	3	AoT
22715750	*Trochalopteron morrisonianum*	EggWidth	NA	3	AoT
22716711	*Heterophasia auricularis*	BodyLength	2	1	ASIZ
22716711	*Heterophasia auricularis*	BodyMass	64	1	AoT; MAPS
22716711	*Heterophasia auricularis*	HeadLength	46	1	ASIZ; MAPS
22716711	*Heterophasia auricularis*	TailLength	68	1	AoT; ASIZ; MAPS
22716711	*Heterophasia auricularis*	TarsusLength	59	1	AoT; ASIZ; MAPS
22716711	*Heterophasia auricularis*	WingLength	59	1	AoT; ASIZ; MAPS
22716711	*Heterophasia auricularis*	ClutchSize	NA	NA	NA
22716711	*Heterophasia auricularis*	IncuPeriod	NA	NA	NA
22716711	*Heterophasia auricularis*	EggLength	NA	NA	NA
22716711	*Heterophasia auricularis*	EggWidth	NA	NA	NA
22715775	*Liocichla steerii*	BodyLength	10	1	ASIZ
22715775	*Liocichla steerii*	BodyMass	335	1	AoT; ASIZ; MAPS
22715775	*Liocichla steerii*	HeadLength	242	1	ASIZ; MAPS
22715775	*Liocichla steerii*	TailLength	306	1	AoT; ASIZ; MAPS
22715775	*Liocichla steerii*	TarsusLength	261	1	AoT; ASIZ; MAPS
22715775	*Liocichla steerii*	WingLength	338	1	AoT; ASIZ; MAPS
22715775	*Liocichla steerii*	ClutchSize	NA	2	AoT
22715775	*Liocichla steerii*	IncuPeriod	NA	3	HBW
22715775	*Liocichla steerii*	EggLength	6	3	AoT
22715775	*Liocichla steerii*	EggWidth	6	3	AoT
22716572	*Sibia morrisoniana*	BodyLength	NA	2	HBW
22716572	*Sibia morrisoniana*	BodyMass	52	1	AoT; MAPS
22716572	*Sibia morrisoniana*	HeadLength	36	1	MAPS
22716572	*Sibia morrisoniana*	TailLength	44	1	AoT; MAPS
22716572	*Sibia morrisoniana*	TarsusLength	45	1	AoT; MAPS
22716572	*Sibia morrisoniana*	WingLength	50	1	AoT; MAPS
22716572	*Sibia morrisoniana*	ClutchSize	NA	NA	NA
22716572	*Sibia morrisoniana*	IncuPeriod	NA	NA	NA
22716572	*Sibia morrisoniana*	EggLength	NA	NA	NA
22716572	*Sibia morrisoniana*	EggWidth	NA	NA	NA
22709223	*Muscicapa ferruginea*	BodyLength	NA	2	HBW
22709223	*Muscicapa ferruginea*	BodyMass	21	1	MAPS
22709223	*Muscicapa ferruginea*	HeadLength	16	1	MAPS
22709223	*Muscicapa ferruginea*	TailLength	29	1	AoT; MAPS
22709223	*Muscicapa ferruginea*	TarsusLength	24	1	AoT; MAPS
22709223	*Muscicapa ferruginea*	WingLength	31	1	AoT; MAPS
22709223	*Muscicapa ferruginea*	ClutchSize	NA	3	AoT
22709223	*Muscicapa ferruginea*	IncuPeriod	NA	NA	NA
22709223	*Muscicapa ferruginea*	EggLength	NA	3	AoT
22709223	*Muscicapa ferruginea*	EggWidth	NA	3	AoT
103756772	*Niltava vivida*	BodyLength	3	1	ASIZ
103756772	*Niltava vivida*	BodyMass	13	1	MAPS
103756772	*Niltava vivida*	HeadLength	15	1	ASIZ; MAPS
103756772	*Niltava vivida*	TailLength	25	1	AoT; ASIZ; MAPS
103756772	*Niltava vivida*	TarsusLength	26	1	AoT; ASIZ; MAPS
103756772	*Niltava vivida*	WingLength	25	1	AoT; ASIZ; MAPS
103756772	*Niltava vivida*	ClutchSize	NA	3	AoT
103756772	*Niltava vivida*	IncuPeriod	NA	NA	NA
103756772	*Niltava vivida*	EggLength	NA	NA	NA
103756772	*Niltava vivida*	EggWidth	NA	NA	NA
103866595	*Brachypteryx montana*	BodyLength	NA	2	HBW
103866595	*Brachypteryx montana*	BodyMass	30	1	AoT; MAPS
103866595	*Brachypteryx montana*	HeadLength	23	1	MAPS
103866595	*Brachypteryx montana*	TailLength	32	1	AoT; MAPS
103866595	*Brachypteryx montana*	TarsusLength	30	1	AoT; MAPS
103866595	*Brachypteryx montana*	WingLength	33	1	AoT; MAPS
103866595	*Brachypteryx montana*	ClutchSize	NA	3	AoT
103866595	*Brachypteryx montana*	IncuPeriod	NA	NA	NA
103866595	*Brachypteryx montana*	EggLength	NA	NA	NA
103866595	*Brachypteryx montana*	EggWidth	NA	NA	NA
22708334	*Myophonus insularis*	BodyLength	14	1	ASIZ
22708334	*Myophonus insularis*	BodyMass	17	1	AoT; ASIZ; MAPS
22708334	*Myophonus insularis*	HeadLength	16	1	ASIZ
22708334	*Myophonus insularis*	TailLength	25	1	AoT; ASIZ; MAPS
22708334	*Myophonus insularis*	TarsusLength	27	1	AoT; ASIZ; MAPS
22708334	*Myophonus insularis*	WingLength	27	1	AoT; ASIZ; MAPS
22708334	*Myophonus insularis*	ClutchSize	6	3	AoT
22708334	*Myophonus insularis*	IncuPeriod	NA	2	HBW
22708334	*Myophonus insularis*	EggLength	4	3	AoT
22708334	*Myophonus insularis*	EggWidth	4	3	AoT
22710123	*Enicurus scouleri*	BodyLength	2	1	ASIZ
22710123	*Enicurus scouleri*	BodyMass	5	1	AoT; MAPS
22710123	*Enicurus scouleri*	HeadLength	3	1	ASIZ; MAPS
22710123	*Enicurus scouleri*	TailLength	13	1	AoT; ASIZ; MAPS
22710123	*Enicurus scouleri*	TarsusLength	13	1	AoT; ASIZ; MAPS
22710123	*Enicurus scouleri*	WingLength	13	1	AoT; ASIZ; MAPS
22710123	*Enicurus scouleri*	ClutchSize	NA	2	HBW
22710123	*Enicurus scouleri*	IncuPeriod	NA	NA	NA
22710123	*Enicurus scouleri*	EggLength	NA	NA	NA
22710123	*Enicurus scouleri*	EggWidth	NA	NA	NA
103768823	*Myiomela leucura*	BodyLength	6	1	ASIZ
103768823	*Myiomela leucura*	BodyMass	413	1	AoT; ASIZ; MAPS
103768823	*Myiomela leucura*	HeadLength	335	1	ASIZ; MAPS
103768823	*Myiomela leucura*	TailLength	385	1	AoT; ASIZ; MAPS
103768823	*Myiomela leucura*	TarsusLength	360	1	AoT; ASIZ; MAPS
103768823	*Myiomela leucura*	WingLength	408	1	AoT; ASIZ; MAPS
103768823	*Myiomela leucura*	ClutchSize	NA	2	AoT
103768823	*Myiomela leucura*	IncuPeriod	NA	NA	NA
103768823	*Myiomela leucura*	EggLength	NA	2	HBW
103768823	*Myiomela leucura*	EggWidth	NA	2	HBW
22709743	*Tarsiger indicus*	BodyLength	NA	2	HBW
22709743	*Tarsiger indicus*	BodyMass	274	1	AoT; MAPS
22709743	*Tarsiger indicus*	HeadLength	187	1	MAPS
22709743	*Tarsiger indicus*	TailLength	255	1	AoT; MAPS
22709743	*Tarsiger indicus*	TarsusLength	206	1	AoT; MAPS
22709743	*Tarsiger indicus*	WingLength	271	1	AoT; MAPS
22709743	*Tarsiger indicus*	ClutchSize	NA	3	AoT
22709743	*Tarsiger indicus*	IncuPeriod	NA	NA	NA
22709743	*Tarsiger indicus*	EggLength	NA	3	AoT
22709743	*Tarsiger indicus*	EggWidth	NA	3	AoT
22709753	*Tarsiger johnstoniae*	BodyLength	3	1	ASIZ
22709753	*Tarsiger johnstoniae*	BodyMass	84	1	AoT; ASIZ; MAPS
22709753	*Tarsiger johnstoniae*	HeadLength	59	1	ASIZ; MAPS
22709753	*Tarsiger johnstoniae*	TailLength	86	1	AoT; ASIZ; MAPS
22709753	*Tarsiger johnstoniae*	TarsusLength	74	1	AoT; ASIZ; MAPS
22709753	*Tarsiger johnstoniae*	WingLength	89	1	AoT; ASIZ; MAPS
22709753	*Tarsiger johnstoniae*	ClutchSize	43	3	AoT
22709753	*Tarsiger johnstoniae*	IncuPeriod	NA	3	AoT
22709753	*Tarsiger johnstoniae*	EggLength	NA	NA	NA
22709753	*Tarsiger johnstoniae*	EggWidth	NA	NA	NA
103769540	*Ficedula hyperythra*	BodyLength	NA	2	HBW
103769540	*Ficedula hyperythra*	BodyMass	90	1	MAPS
103769540	*Ficedula hyperythra*	HeadLength	75	1	MAPS
103769540	*Ficedula hyperythra*	TailLength	89	1	AoT; MAPS
103769540	*Ficedula hyperythra*	TarsusLength	78	1	AoT; MAPS
103769540	*Ficedula hyperythra*	WingLength	93	1	AoT; MAPS
103769540	*Ficedula hyperythra*	ClutchSize	NA	3	AoT
103769540	*Ficedula hyperythra*	IncuPeriod	NA	NA	NA
103769540	*Ficedula hyperythra*	EggLength	NA	3	AoT
103769540	*Ficedula hyperythra*	EggWidth	NA	3	AoT
22710092	*Phoenicurus fuliginosus*	BodyLength	2	1	ASIZ
22710092	*Phoenicurus fuliginosus*	BodyMass	21	1	AoT; ASIZ
22710092	*Phoenicurus fuliginosus*	HeadLength	2	1	ASIZ
22710092	*Phoenicurus fuliginosus*	TailLength	22	1	AoT; ASIZ
22710092	*Phoenicurus fuliginosus*	TarsusLength	22	1	AoT; ASIZ
22710092	*Phoenicurus fuliginosus*	WingLength	22	1	AoT; ASIZ
22710092	*Phoenicurus fuliginosus*	ClutchSize	NA	NA	NA
22710092	*Phoenicurus fuliginosus*	IncuPeriod	NA	3	AoT
22710092	*Phoenicurus fuliginosus*	EggLength	NA	3	AoT
22710092	*Phoenicurus fuliginosus*	EggWidth	NA	3	AoT
22708286	*Monticola solitarius*	BodyLength	5	1	ASIZ
22708286	*Monticola solitarius*	BodyMass	19	1	AoT; ASIZ
22708286	*Monticola solitarius*	HeadLength	6	1	ASIZ
22708286	*Monticola solitarius*	TailLength	23	1	AoT; ASIZ
22708286	*Monticola solitarius*	TarsusLength	23	1	AoT; ASIZ
22708286	*Monticola solitarius*	WingLength	24	1	AoT; ASIZ
22708286	*Monticola solitarius*	ClutchSize	NA	2	HBW
22708286	*Monticola solitarius*	IncuPeriod	NA	2	HBW
22708286	*Monticola solitarius*	EggLength	NA	NA	NA
22708286	*Monticola solitarius*	EggWidth	NA	NA	NA
103879357	*Zoothera dauma*	BodyLength	9	1	ASIZ
103879357	*Zoothera dauma*	BodyMass	6	1	ASIZ; MAPS
103879357	*Zoothera dauma*	HeadLength	12	1	ASIZ; MAPS
103879357	*Zoothera dauma*	TailLength	13	1	AoT; ASIZ; MAPS
103879357	*Zoothera dauma*	TarsusLength	14	1	AoT; ASIZ; MAPS
103879357	*Zoothera dauma*	WingLength	14	1	AoT; ASIZ; MAPS
103879357	*Zoothera dauma*	ClutchSize	NA	3	HBW
103879357	*Zoothera dauma*	IncuPeriod	NA	NA	NA
103879357	*Zoothera dauma*	EggLength	NA	NA	NA
103879357	*Zoothera dauma*	EggWidth	NA	NA	NA
103888237	*Turdus mandarinus*	BodyLength	1	1	ASIZ
103888237	*Turdus mandarinus*	BodyMass	4	1	AoT; ASIZ; MAPS
103888237	*Turdus mandarinus*	HeadLength	3	1	ASIZ; MAPS
103888237	*Turdus mandarinus*	TailLength	4	1	AoT; ASIZ; MAPS
103888237	*Turdus mandarinus*	TarsusLength	4	1	AoT; ASIZ; MAPS
103888237	*Turdus mandarinus*	WingLength	5	1	AoT; ASIZ; MAPS
103888237	*Turdus mandarinus*	ClutchSize	NA	2	HBW
103888237	*Turdus mandarinus*	IncuPeriod	NA	2	HBW
103888237	*Turdus mandarinus*	EggLength	NA	NA	NA
103888237	*Turdus mandarinus*	EggWidth	NA	NA	NA
103891993	*Turdus poliocephalus*	BodyLength	NA	2	HBW
103891993	*Turdus poliocephalus*	BodyMass	13	1	AoT; MAPS
103891993	*Turdus poliocephalus*	HeadLength	11	1	MAPS
103891993	*Turdus poliocephalus*	TailLength	20	1	AoT; MAPS
103891993	*Turdus poliocephalus*	TarsusLength	23	1	AoT; MAPS
103891993	*Turdus poliocephalus*	WingLength	21	1	AoT; MAPS
103891993	*Turdus poliocephalus*	ClutchSize	NA	2	AoT
103891993	*Turdus poliocephalus*	IncuPeriod	NA	3	HBW
103891993	*Turdus poliocephalus*	EggLength	NA	3	AoT
103891993	*Turdus poliocephalus*	EggWidth	NA	3	AoT
22710896	*Spodiopsar cineraceus*	BodyLength	NA	2	HBW
22710896	*Spodiopsar cineraceus*	BodyMass	NA	2	HBW
22710896	*Spodiopsar cineraceus*	HeadLength	NA	NA	NA
22710896	*Spodiopsar cineraceus*	TailLength	NA	NA	NA
22710896	*Spodiopsar cineraceus*	TarsusLength	NA	NA	NA
22710896	*Spodiopsar cineraceus*	WingLength	NA	NA	NA
22710896	*Spodiopsar cineraceus*	ClutchSize	NA	2	HBW
22710896	*Spodiopsar cineraceus*	IncuPeriod	NA	2	HBW
22710896	*Spodiopsar cineraceus*	EggLength	NA	NA	NA
22710896	*Spodiopsar cineraceus*	EggWidth	NA	NA	NA
22710946	*Acridotheres cristatellus*	BodyLength	3	1	ASIZ
22710946	*Acridotheres cristatellus*	BodyMass	NA	2	HBW
22710946	*Acridotheres cristatellus*	HeadLength	3	1	ASIZ
22710946	*Acridotheres cristatellus*	TailLength	13	1	AoT; ASIZ
22710946	*Acridotheres cristatellus*	TarsusLength	13	1	AoT; ASIZ
22710946	*Acridotheres cristatellus*	WingLength	13	1	AoT; ASIZ
22710946	*Acridotheres cristatellus*	ClutchSize	NA	2	AoT
22710946	*Acridotheres cristatellus*	IncuPeriod	NA	3	HBW
22710946	*Acridotheres cristatellus*	EggLength	NA	2	AoT
22710946	*Acridotheres cristatellus*	EggWidth	NA	2	AoT
103776140	*Dicaeum minullum*	BodyLength	8	1	YIO
103776140	*Dicaeum minullum*	BodyMass	1	1	MAPS
103776140	*Dicaeum minullum*	HeadLength	9	1	MAPS; YIO
103776140	*Dicaeum minullum*	TailLength	10	1	AoT; MAPS; YIO
103776140	*Dicaeum minullum*	TarsusLength	2	1	AoT; MAPS
103776140	*Dicaeum minullum*	WingLength	10	1	AoT; MAPS; YIO
103776140	*Dicaeum minullum*	ClutchSize	NA	2	HBW
103776140	*Dicaeum minullum*	IncuPeriod	NA	NA	NA
103776140	*Dicaeum minullum*	EggLength	NA	NA	NA
103776140	*Dicaeum minullum*	EggWidth	NA	NA	NA
103776843	*Dicaeum ignipectus*	BodyLength	7	1	YIO
103776843	*Dicaeum ignipectus*	BodyMass	1	1	MAPS
103776843	*Dicaeum ignipectus*	HeadLength	8	1	MAPS; YIO
103776843	*Dicaeum ignipectus*	TailLength	12	1	AoT; MAPS; YIO
103776843	*Dicaeum ignipectus*	TarsusLength	5	1	AoT; MAPS
103776843	*Dicaeum ignipectus*	WingLength	11	1	AoT; MAPS; YIO
103776843	*Dicaeum ignipectus*	ClutchSize	NA	2	AoT
103776843	*Dicaeum ignipectus*	IncuPeriod	NA	NA	NA
103776843	*Dicaeum ignipectus*	EggLength	NA	2	HBW
103776843	*Dicaeum ignipectus*	EggWidth	NA	2	HBW
22718617	*Prunella collaris*	BodyLength	9	1	YIO
22718617	*Prunella collaris*	BodyMass	NA	2	HBW
22718617	*Prunella collaris*	HeadLength	9	1	YIO
22718617	*Prunella collaris*	TailLength	11	1	AoT; YIO
22718617	*Prunella collaris*	TarsusLength	11	1	AoT; YIO
22718617	*Prunella collaris*	WingLength	11	1	AoT; YIO
22718617	*Prunella collaris*	ClutchSize	NA	2	HBW
22718617	*Prunella collaris*	IncuPeriod	NA	2	HBW
22718617	*Prunella collaris*	EggLength	NA	NA	NA
22718617	*Prunella collaris*	EggWidth	NA	NA	NA
22718348	*Motacilla alba*	BodyLength	6	1	ASIZ
22718348	*Motacilla alba*	BodyMass	2	1	ASIZ; MAPS
22718348	*Motacilla alba*	HeadLength	7	1	ASIZ; MAPS
22718348	*Motacilla alba*	TailLength	13	1	AoT; ASIZ; MAPS
22718348	*Motacilla alba*	TarsusLength	13	1	AoT; ASIZ; MAPS
22718348	*Motacilla alba*	WingLength	13	1	AoT; ASIZ; MAPS
22718348	*Motacilla alba*	ClutchSize	NA	2	AoT
22718348	*Motacilla alba*	IncuPeriod	NA	2	HBW
22718348	*Motacilla alba*	EggLength	NA	3	AoT
22718348	*Motacilla alba*	EggWidth	NA	3	AoT
103837801	*Pyrrhula nipalensis*	BodyLength	10	1	ASIZ; YIO
103837801	*Pyrrhula nipalensis*	BodyMass	14	1	ASIZ; MAPS
103837801	*Pyrrhula nipalensis*	HeadLength	19	1	MAPS; YIO
103837801	*Pyrrhula nipalensis*	TailLength	25	1	AoT; ASIZ; MAPS: YIO
103837801	*Pyrrhula nipalensis*	TarsusLength	25	1	AoT; ASIZ; MAPS: YIO
103837801	*Pyrrhula nipalensis*	WingLength	25	1	AoT; ASIZ; MAPS: YIO
103837801	*Pyrrhula nipalensis*	ClutchSize	NA	NA	NA
103837801	*Pyrrhula nipalensis*	IncuPeriod	NA	NA	NA
103837801	*Pyrrhula nipalensis*	EggLength	NA	NA	NA
103837801	*Pyrrhula nipalensis*	EggWidth	NA	NA	NA
22720668	*Pyrrhula erythaca*	BodyLength	9	1	ASIZ; YIO
22720668	*Pyrrhula erythaca*	BodyMass	12	1	MAPS
22720668	*Pyrrhula erythaca*	HeadLength	21	1	ASIZ; MAPS: YIO
22720668	*Pyrrhula erythaca*	TailLength	20	1	AoT; ASIZ; MAPS
22720668	*Pyrrhula erythaca*	TarsusLength	27	1	AoT; ASIZ; MAPS: YIO
22720668	*Pyrrhula erythaca*	WingLength	28	1	AoT; ASIZ; MAPS: YIO
22720668	*Pyrrhula erythaca*	ClutchSize	NA	3	HBW
22720668	*Pyrrhula erythaca*	IncuPeriod	NA	NA	NA
22720668	*Pyrrhula erythaca*	EggLength	NA	NA	NA
22720668	*Pyrrhula erythaca*	EggWidth	NA	NA	NA
103834794	*Carpodacus formosanus*	BodyLength	1	1	ASIZ
103834794	*Carpodacus formosanus*	BodyMass	47	1	MAPS
103834794	*Carpodacus formosanus*	HeadLength	44	1	ASIZ; MAPS
103834794	*Carpodacus formosanus*	TailLength	56	1	AoT; ASIZ; MAPS
103834794	*Carpodacus formosanus*	TarsusLength	54	1	AoT; ASIZ; MAPS
103834794	*Carpodacus formosanus*	WingLength	58	1	AoT; ASIZ; MAPS
103834794	*Carpodacus formosanus*	ClutchSize	NA	NA	NA
103834794	*Carpodacus formosanus*	IncuPeriod	NA	NA	NA
103834794	*Carpodacus formosanus*	EggLength	NA	NA	NA
103834794	*Carpodacus formosanus*	EggWidth	NA	NA	NA
22718191	*Passer cinnamomeus*	BodyLength	7	1	YIO
22718191	*Passer cinnamomeus*	BodyMass	NA	2	HBW
22718191	*Passer cinnamomeus*	HeadLength	7	1	YIO
22718191	*Passer cinnamomeus*	TailLength	7	1	YIO
22718191	*Passer cinnamomeus*	TarsusLength	7	1	YIO
22718191	*Passer cinnamomeus*	WingLength	7	1	YIO
22718191	*Passer cinnamomeus*	ClutchSize	NA	2	HBW
22718191	*Passer cinnamomeus*	IncuPeriod	NA	NA	NA
22718191	*Passer cinnamomeus*	EggLength	NA	NA	NA
22718191	*Passer cinnamomeus*	EggWidth	NA	NA	NA
22718270	*Passer montanus*	BodyLength	12	1	ASIZ
22718270	*Passer montanus*	BodyMass	9	1	ASIZ
22718270	*Passer montanus*	HeadLength	15	1	ASIZ
22718270	*Passer montanus*	TailLength	25	1	AoT; ASIZ
22718270	*Passer montanus*	TarsusLength	25	1	AoT; ASIZ
22718270	*Passer montanus*	WingLength	25	1	AoT; ASIZ
22718270	*Passer montanus*	ClutchSize	NA	2	AoT
22718270	*Passer montanus*	IncuPeriod	NA	2	HBW
22718270	*Passer montanus*	EggLength	NA	3	AoT
22718270	*Passer montanus*	EggWidth	NA	3	AoT
22719806	*Lonchura striata*	BodyLength	2	1	ASIZ
22719806	*Lonchura striata*	BodyMass	68	1	MAPS
22719806	*Lonchura striata*	HeadLength	68	1	ASIZ; MAPS
22719806	*Lonchura striata*	TailLength	73	1	AoT; ASIZ; MAPS
22719806	*Lonchura striata*	TarsusLength	81	1	AoT; ASIZ; MAPS
22719806	*Lonchura striata*	WingLength	82	1	AoT; ASIZ; MAPS
22719806	*Lonchura striata*	ClutchSize	NA	2	HBW
22719806	*Lonchura striata*	IncuPeriod	NA	3	HBW
22719806	*Lonchura striata*	EggLength	NA	NA	NA
22719806	*Lonchura striata*	EggWidth	NA	NA	NA
22719821	*Lonchura punctulata*	BodyLength	11	1	ASIZ
22719821	*Lonchura punctulata*	BodyMass	26	1	ASIZ; MAPS
22719821	*Lonchura punctulata*	HeadLength	35	1	ASIZ; MAPS
22719821	*Lonchura punctulata*	TailLength	44	1	AoT; ASIZ; MAPS
22719821	*Lonchura punctulata*	TarsusLength	47	1	AoT; ASIZ; MAPS
22719821	*Lonchura punctulata*	WingLength	48	1	AoT; ASIZ; MAPS
22719821	*Lonchura punctulata*	ClutchSize	NA	3	AoT
22719821	*Lonchura punctulata*	IncuPeriod	NA	2	AoT
22719821	*Lonchura punctulata*	EggLength	NA	3	AoT
22719821	*Lonchura punctulata*	EggWidth	NA	3	AoT
22729138	*Lonchura atricapilla*	BodyLength	NA	2	HBW
22729138	*Lonchura atricapilla*	BodyMass	NA	2	HBW
22729138	*Lonchura atricapilla*	HeadLength	NA	NA	NA
22729138	*Lonchura atricapilla*	TailLength	2	1	AoT
22729138	*Lonchura atricapilla*	TarsusLength	2	1	AoT
22729138	*Lonchura atricapilla*	WingLength	2	1	AoT
22729138	*Lonchura atricapilla*	ClutchSize	NA	2	HBW
22729138	*Lonchura atricapilla*	IncuPeriod	NA	3	HBW
22729138	*Lonchura atricapilla*	EggLength	NA	NA	NA
22729138	*Lonchura atricapilla*	EggWidth	NA	NA	NA

* Calculation methods: 1 - the mean and/or standard deviation of a trait value were calculated from the measurements of individual birds, broods or eggs; 2 - the mean of a trait value was calculated as the average of the maximum and minimum trait values obtained from the data source; 3 - the mean and/or standard deviation of a trait value were obtained directly from the data source.

# Data sources: AoT - the Avifauna of Taiwan; ASIZ - Biodiversity Research Museum of Academia Sinica; Elton - EltonTraits dataset; HBW - the Handbook of the Birds of the World; MAPS - MAPS Taiwan banding dataset; TM - National Taiwan Museum; and YIO - Yamashina Institute for Ornithology.
